# Maternal probiotic exposure enhances CD8 T cell protective neonatal immunity and modulates offspring metabolome to control influenza virus infection

**DOI:** 10.1080/19490976.2024.2442526

**Published:** 2024-12-22

**Authors:** Clara Valentin, Patricia Brito Rodrigues, Marko Verce, Sandrine Delbauve, Léa La Palombara, Florine Demaret, Justine Allard, Isabelle Salmon, Patrice D. Cani, Arnaud Köhler, Amandine Everard, Véronique Flamand

**Affiliations:** a Institute for Medical Immunology, Université Libre de Bruxelles, Gosselies, Belgium; b ULB Center for Research in Immunology (U-CRI), Gosselies, Belgium; c Metabolism and Nutrition Research Group, Louvain Drug Research Institute, UCLouvain, Université Catholique de Louvain, Brussels, Belgium; d Walloon Excellence in Life Sciences and BIOtechnology (WELBIO) Department, WEL Research Institute, Wavre, Belgium; e DIAPath, Center for Microscopy and Molecular Imaging, Université Libre de Bruxelles, Gosselies, Belgium; f Institute of Experimental and Clinical Research (IREC), UCLouvain, Université Catholique de Louvain, Brussels, Belgium

**Keywords:** IAV, CD8 T cells, early life, memory response, *Lacticaseibacillus rhamnosus*, *Bifidobacterium*

## Abstract

Maternal gut microbiota composition contributes to the status of the neonatal immune system and could influence the early life higher susceptibility to viral respiratory infections. Using a novel protocol of murine maternal probiotic supplementation, we report that perinatal exposure to *Lacticaseibacillus rhamnosus* (*L.rh*) or *Bifidobacterium animalis subsp. lactis* (*B.lac*) increases the influenza A/PR8 virus (IAV) clearance in neonates. Following either supplementation, type 1 conventional dendritic cells (cDC1) were amplified in the lymph nodes leading to an enhanced IAV antigen-experienced IFN-γ producing effector CD8 T cells in neonates and IAV-specific resident memory CD8 T cells in adulthood. This was compatible with a higher protection of the offspring upon a secondary infection. Interestingly, only mice born to *L.rh* supplemented mothers further displayed an increased activation of IFN-γ producing virtual memory CD8 T cells and a production of IL-10 by CD4 and CD8 T cells that could explain a better control of the lung damages upon infection. In the offspring and the mothers, no disturbance of the gut microbiota was observed but, as analyzed through an untargeted metabolomic approach, both exposures modified neonatal plasma metabolites. Among them, we further demonstrated that genistein and 3-(3-hydroxyphenyl)propionic acid recapitulate viral clearance or cDC1 activation in neonates exposed to IAV. We conclude that maternal *L.rh* or *B.lac* supplementation confers the neonates specific metabolomic modulations with a better CD8 T cell-mediated immune protection against IAV infection.

## Introduction

Influenza viruses are responsible for seasonal epidemics and have caused six pandemics in the past 140 years. The symptoms of influenza, commonly known as the “flu”, include fever, cough, sore throat, body aches, and fatigue. While most people recover within a week without specific treatment, those in “high-risk” population may develop fatal pneumonia. According to the WHO, the influenza virus causes worldwide 290.000 to 650.000 respiratory deaths annually.^1^ High-risk groups include the elderly (65+), individuals with chronic medical conditions or immunocompromised, pregnant women, and children under 5-years old. To prevent this population to develop severe symptoms of the lower respiratory tract, vaccinations have been used for more than 60 y. Nevertheless, neonates under 6 months of age cannot benefit from this protection.

The increased susceptibility of newborns to infections was first explained by the neonatal T-cell tolerance, concept from the 1980s, which suggested an impaired T-cell immunity development with a lack of memory responses.^2^ Over the years, scientists documented that the perinatal immune system undergoes a stratified development of innate and adaptive immune cells with unique specialized capacities. These capacities are adapted to the needs of the host^3,4^ promoting tolerance against harmless non-inherited maternal antigens and supporting microbiota colonization.^5^ As part of this, the particular features of the neonatal system are preferential Th2 and Treg-biased responses instead of potential harmful Th1-linked inflammation.^4,6^ During the pathogenic respiratory influenza virus infections, neonates displayed a defective expansion of effector T cells. Compared to adults, they mount delayed and less robust IFN-γ producing Th1 and cytotoxic CD8 T lymphocytes (CTL) responses which are crucial for clearance of the virus.^7,8^

Accumulating evidence have highlighted the importance of the first exposure to commensals that shape the microbiota and modulate the development of the immune system with disease risk impacts throughout life.^9,10^ Notably, a unique and limited period called the “early life window of variation” has been identified in murine and human neonates. During this period, profound alterations of the gut microbiota can modulate the immune protection.^11^ We demonstrated in murine neonates that microbiota deprivation during this critical period delays the development of type 1 conventional dendritic cells (cDC1s), which negatively impacts on the intensity of the protective IFN-γ^+^ CD8 T cell response against *Listeria monocytogenes* infection.^12^ Other studies were focused on the crosstalk between the gut microbiota and the lungs termed as the gut-lung axis. Several factors that can affect the composition of the intestinal microbiota like the mode of delivery, the maternal and/or neonatal medical treatments (antibiotics), the diet, and the diseases influence the immune response and the homeostasis of the lungs of the offspring. These microbial changes may have a long-lasting impact on their immune system by increasing their susceptibility to developing lung diseases such as asthma and severe pathology upon exposure to pathogens such as the Influenza A virus (IAV).^13–15^ Knowing the protective role of a healthy gut microbiota, new approaches have been proposed involving the use of probiotics to regulate the immune system and to prevent respiratory diseases. According to the WHO, probiotics are “live microorganisms which when administered in adequate amounts confer a health benefit on the host”.^16^ Several studies have identified specific strains of probiotics mainly from the former genus *Lactobacillus (Lacticaseibacillus paracasei, Lacticaseibacillus rhamnosus, Levilactobacillus brevis*) and from the genus *Bifidobacterium (B. animalis)* that could help to prevent or reduce the severity of respiratory diseases such as IAV in adult mice and in humans.^17–20^ However, these benefits could be strain-specific, as shown by the variation in efficacy across meta-analyses.^21^ Moreover, very few studies have focused their attention on the neonatal/childhood period where the microbiota starts to develop, to understand the benefit of probiotics on the IAV infection. In neonatal mice, intranasal (i.n.) administration of probiotics has been shown to prevent in a type I IFN-dependent manner unresponsiveness and loss of early infection control.^22^ In humans, only few studies have observed the benefit of probiotics treatments to reduce school absenteeism and disease duration.^23,24^ Despite these findings, no studies have yet explored the potential benefit of administering probiotics to pregnant/breastfeeding mothers to modulate the offspring adaptive T cell responses and protect them against a viral infection.

Here we investigated the effects of two probiotic strains, *Lacticaseibacillus rhamnosus* VES 001 (*L.rh*) previously demonstrated as effectively preventing Th-2 type response in murine neonates^25^ and *Bifidobacterium animalis subsp. lactis* VES 002 (*B.lac*). Each strain was administered separately to pregnant and breastfeeding mothers and the effects on the neonatal immune response against an IAV infection were evaluated. We focused on key disease parameters and the induction of CD8 T cell neonatal responses against the infection. Moreover, we performed 16S rRNA sequencing and metabolomic analysis in neonates to obtain a deeper insight into the mechanisms potentially involved in the probiotic effects.

## Materials and methods

### Mice

C57BL/6 mice were purchased from ENVIGO (Zeist, Tthe Netherlands). Mice were housed and bred in our specific pathogen-free animal facility in individually ventilated cages with a controlled day-night cycle and given food and water ad libitum. All experiments were approved by the institutional Animal Care and Local Use Committee. During the experimental time, mice were housed in the same conditions in a Biosecurity Level 2 infectious animal facility.

### Maternal probiotic supplementation

The 15th day of gestation (i.e. 6 d before delivery), pregnant C57BL/6 females (10–12 weeks) were orally administered daily by gavage with 2 × 10^8^ CFU *Lacticaseibacillus rhamnosus* VES 001 (*L.rh*) (SUPERBIOTICS, Eghezée, Belgium) or 2 × 10^8^ CFU *Bifidobacterium animalis subsp. lactis* VES 002 (*B.lac*) (SUPERBIOTICS, Eghezée, Belgium)/100 µl of NaCl 0.9% (B. Braun, Melsungen, Deutschland) as previously described.^25^ The treatment was maintained until three-day post-delivery at the rate of one daily gavage.

### Neonatal metabolite supplementation

On their second and third day of life, neonates were intraperitoneally injected with 20 mg/kg of cinnamoylglycine (bioconnect, Huissen, The Netherlands), genistein (Merck, Darmstadt, Germany) or 3-(3-hydroxyphenyl)propionic acid (Sanbio, Uden, The Netherlands) diluted into DMSO (0,8%). Two hours after the last injection, neonates were infected with the virus as described in the Influenza A infectious model section.

### Bacteria DNA collection

Intestinal content samples were collected from all experimental groups (CTRL, *B.lac*, and *L.rh*) at different time points from the neonates (day 1, 3, and 7 post-delivery), as well as from the mothers. Neonate samples were pooled by litter. Metagenomic DNA was extracted using a protocol based on the QIAamp DNA Stool Mini Kit (Qiagen, Hilden, Germany). The V3–V4 region of the 16S rRNA gene was amplified using the primers rambacV3F (5'-CCTACGGGAGGCAGCAG-3') and rambacV4R (5'-GGACTACHVGGGTWTCTAAT-3'), followed by sequencing using an Illumina MiSeq platform at MR DNA (www.mrdnalab.com, Shallowater, TX, USA). Samples were sequenced along with an elution buffer control in two runs per probiotic trial and sequence reads were processed using QIIME2 (q2cli 2021.4.0).^26^ To ensure sequence quality, only forward sequencing reads were used for the analysis. Cutadapt^27^ was used to remove primers and DADA2 for denoising.^28^ The following DADA2 parameters were used: maximum expected error = 2, truncation length = 232 nt, minimum fold parent over-abundance = 4. The sequencing data were submitted to the European Nucleotide Archive (ENA/EBI) and are available under the study accession number PRJEB72811.

### Gut microbiota analysis

For the gut microbiota analysis, diversity metrics were calculated in QIIME2. Based on the alpha-diversity rarefaction curves, the minimum sample sizes of 11,908 and 13,699 reads for the *L.rh* mother and neonate data sets, respectively, and 20,039 and 12,652 reads for the *B.lac* mother and neonate data sets, respectively. As a result, 3 samples were excluded from the *L.rh* (2 from neonate, and 1 from the mother group) data set analysis and one sample from the *B.lac* (1 from neonate group) data set analysis. PCoA was performed using the weighted and unweighted UniFrac distances (wUniFrac and unwUniFrac, respectively) with QIIME2. Taxonomy was assigned to ASVs using a classifier trained on the database SILVA 138.1 SSURef NR99,^29^ trimmed to the V3-V4 region using RESCRIPt.^30^ To assess differential abundance between groups on the level of ASVs and the lowest taxonomic level down to genus, the compositional data analysis tools ANCOM-BC^31^ and ALDEx2^32^were used. When listing the ASVs, their names were simplified to be composed of the name of the assigned genus (or a higher taxon if genus not possible) and ASVx, whereby × represents the rank among all ASVs belonging to this taxon, when sorted by mean relative frequency in the data set. The PCoA and taxonomy were visualized using the “tidyverse”^33^ collection of R packages. Differences in alpha-diversity metrics of gut microbiota samples (Pielou’s evenness in rarefied samples) were assessed using the Kruskal –Wallis test, followed by Dunn’s test for pairwise differences, or using the aligned rank transformation ANOVA (ART ANOVA)^34^ the R package ARTool^35^ in the case of two-way statistical testing. PERMANOVA/Adonis^36^ and PERMDISP with 9999 permutations were used in QIIME2 to assess differences in beta-diversity of gut microbiota samples. The p-values were adjusted using the Benjamini–Hochberg procedure where applicable and adjusted p-values are denoted as q.

### Influenza A infectious model

The viral stock was created in our laboratory from the infection of Madin-Darby canine kidney cells (MDCKs; ATCC). The viral stock was stored in aliquots at −80°C and when defrost the rest of the aliquot was discarded to avoid freeze-defreeze cycles. Before infecting mice for the experiments, the LD50 of the viral batch was determined on neonates (nLD50) and on young mice (LD50). Three-day-old mice, slightly anesthetized with inhaled isoflurane, were infected intranasally (i.n.) with 0.5 × nLD50 of the influenza A virus H1N1 strain PR8 (A/Puerto Rico/8/34; ATCC, Manassas, Virginia) in a 5 µl volume (adapted from Kumova et al., 2019).^22^ For the young murine model of IAV, mice between 5 and 6 weeks were slightly anesthetized with inhaled isoflurane before receiving 0.5 × LD50 of IAV in a 20 µl volume (adapted from Kumova et al., 2019).^22^ Analyses of the infected lungs were done at 3-6-9 dpi in neonates and at 30 dpi and 3/6 days post-recall in young mice.

### Influenza A replication

The lungs of the neonates or young mice were harvested, weighted and frozen at −80°C. RNA was isolated from the entire lungs by the Nucleospin^TM^ RNA Plus kit (Macherey Nagel) according to the manufacturer’s protocol. RNA samples were quantified using the Nanodrop^TM^ spectrometer and stored at −20°C. Then, we performed a RT-qPCR with the RNA virus master kit (Roche) using the PR8 (A/Puerto Rico/8/34) specific primers: 4 µl of RT qPCR mix n° 2 (5×), 0,1 µl of RT enzyme solution n° 1 (200×), 1 µl of influenza A virus sense primer (6 µM) (5’-AAGACCAATCCTGTCACCTCTGA-3’), 1 µl of influenza A virus antisense primer (6 µM) (5’-CAAAGACCAATCCTGTCAGTCC-3’) and 1 µl of influenza A virus probe (4 µM) (FAM-5’-TTTGTGTTCACGCTCACCGT-3’-TAMRA) on the Lightcycler® 480 apparatus (Roche). For viral load measurement, a standard curve was developed with a serial 10-fold dilution of PR8 stock with a known Plate forming Units (PFU) concentration. Ct values are represented by virus quantity in PFU per milliliter. The curve was used to transform the Ct values for viral loads to PFU equivalents. Virus RNA quantities in lungs were expressed as PFU equivalents/100 mg of lungs.

### Real-time quantitative PCR from frozen tissues

Lungs were harvested, weighted, and frozen at −80°C. RNA was extracted using the RNA Plus NucleoSpin® following the manufacturer’s protocol (Machery-Nagel; Düren, Germany). Then, we performed an RT-qPCR with the RNA virus master kit (Roche, Basel, Switzerland) on the Lightcycler® 480 equipment (Roche, Machelen, Belgium). RNA quantification was obtained using the NanoDrop 1000 Spectrophotometer (Thermo Scientific) and samples were all diluted to work with a quantity of 100 ng/sample. Moreover, Hypoxanthine Phosphoribosyltransferase (HPRT) and Peptidylprolyl isomerase A (PPIA) were used as housekeeping genes to normalize mRNA levels between samples. The list of designed primers for the detection cytokines/chemokines can be found in the Supplementary materials Table S1. For individual samples, relative RNA levels (2–ΔΔCt) were determined by comparing a) the cycle thresholds (Ct) for the gene of interest and calibrator gene (ΔCt), the geomean of HPRT and PPIA and b) 2–ΔCt values for the experimental group vs. the reference sample (2–ΔCt values mean of the non-infected neonates at 3-days-old or 38-days-old young mice).

### Metabolomic analysis

Plasma and the milk inside the stomachs of the neonates were collected from all experimental groups (CTRL, *B.lac* and *L.rh*) from the neonates at 3 d post-delivery. At the same moment, we euthanized the mothers and collected the plasma. Untargeted metabolomes were analyzed using Ultrahigh Performance Liquid Chromatography-Tandem Mass Spectroscopy (UPLC-MS/MS) by Metabolon (Durham, USA). Briefly, all the samples were maintained at −80°C until processed. Samples were prepared using the MicroLab STAR® system from the Hamilton Company. Recovery standards were added for quality control before the extraction process. To eliminate proteins and recover diverse metabolites, proteins were precipitated with methanol and centrifuged. After the extraction, samples were briefly placed on a TurboVap® to remove organic solvent, and the extracts were stored overnight under nitrogen before analysis preparation. Four aliquots of the sample were analyzed using different UPLC-MS/MS conditions. One aliquot (PosEarly) was analyzed under acidic positive ion conditions with a C18 column, optimized for hydrophilic compounds. Another aliquot (PosLate) under the same conditions but optimized for hydrophobic compounds, using a higher overall organic content. The third aliquot (Neg) was analyzed using basic negative ion optimized conditions with a dedicated C18 column. The fourth aliquot was analyzed via negative ionization using a HILIC column. The MS analysis involved alternating between MS and data-dependent MSn scans with dynamic exclusion, covering a scan range of 70–1000 m/z.^37^

### Metabolites identification and analysis

Raw data was extracted, peak-identified and QC processed using a combination of Metabolon-developed software services (applications). Identification criteria included retention index, accurate mass match, and MS/MS scores. Peaks were quantified using area-under-the-curve. A total of 30 samples of offspring’s plasma, 30 samples of mother’s plasma and 30 milk samples were analyzed (*n* = 10/group). The current plasma datasets consist of 1049 features, with 932 known compounds, and 117 being unknown. The milk dataset consists of 822 features, with 759 known compounds, and 63 being unknown. Subsequently, data processing and statistical analysis were conducted using MetaboAnalyst (www.metaboanalyst.ca), separately for each type of sample and origin (mother or offspring). Unsupervised analysis (PCA) was performed and revealed one *L.rh* sample as a strong outlier (data not shown). After excluding the outlier, the data matrix was divided into two subsets based on chromatographic analytical platforms.^38,39^ PosEarly and HILIC were considered to create the hydrophilic matrix, while PosLate and Negative were used for the hydrophobic matrix. Data analyses for each matrix were conducted in the following four steps: 1) Missing values were filtered (threshold of 70%) and imputed by 1/5 of the minimum positive value, followed by normalization using the sample median, Log10 transformation, and autoscaling; 2) Multivariate regression evaluation was performed by Partial least squares discriminant analysis (PLS-DA); 3) To verify the statistical significance of the distance between the cluster of samples visualized on the PLS-DA score plot, model validation by permutation tests was tested; and 4) To access a preliminary overview about features that are potentially significant in discriminating the conditions under study, the univariate analyses was applied by one-way Analysis of Variance (ANOVA); p-value (FDR) <0.05 cutoff; Post-hoc analysis by Tukey’s Honestly Significant Difference (HSD) with raw p-value cutoff <0.05. All significant metabolites of offspring plasma discovered by one-way ANOVA from hydrophilic and hydrophobic data sets were compiled, and the fold change (FC) value for each significant metabolites was determined by dividing the average signal abundance of the *B.lac* or *L.rh* by the average value of the control group (Supplementary materials Table S2). Hierarchical cluster analysis and heatmap and pathway analysis (based on the Small Molecule Pathway Database – SMPDB), and classification of important features by Random Forest were performed using this compilation of significant metabolites. The heatmap was created using Phantasus v 1.21.5 (https://ctlab.itmo.ru/phantasus.). The summary about the percentages of significant metabolites from each Tukey’s comparison result with *p* < 0.05 was generated and plotted using R (4.3.1) packages dplyr^40^ and ggplot2.^41^

### Histopathology

Neonates or young adults were infected with 0.5 × nLD50 or 0.75 × LD50, respectively. Lungs were harvested at 6 dpi for the neonates and 5 dpi for the young adults. Lung tissues were fixed in neutral buffered formalin solution, dehydrated, and embedded in paraffin. Paraffin sections (4-µm thickness) were cut, dewaxed, and stained with hematoxylin–eosin (HE) for histopathologic evaluation under light microscopy. HE slides were scored by a pathologist (I.S.) in a blinded manner on the basis of the following parameters: necrotizing bronchiolitis, alveolitis, hemorrhage, and edema (based on Narasaraju T *et al*.).^42^ The severity of damage was scored using a four-tiered scoring system as follows: 0, none or very minor; 1, mild; 2, intermediate; 3, moderately severe; and 4, severe and widespread. The highest score observed in the lung samples was considered regardless of extent. In order to take into account such an heterogeneity, a quantitative lesion foci measurement was performed using NDP.view2 software (Hamamatsu-City, Japan) according to $\left(\frac{\mathrm{Total}\;\mathrm{pneuminia}\;\mathrm{surfaces}}{\mathrm{Total}\;\mathrm{lungs}\;\mathrm{surfaces}}\right)X\;100$ (J.A, C.V, I.S). Whole slide imaging was performed using a NanoZoomer S360 (x40 magnification, 230 nm/pixel, Hamamatsu, Hamamatsu-City, Japan). The inducible bronchus-associated lymphoid tissues (iBALTs) were identified by C.V and counted on the total lung surface using the NDP.view2 software (Hamamatsu, Hamamatsu-City, Japan). Finally, their percentage area were calculated thanks to the same software with the following calculus: $\left(\frac{\mathrm{Total}\;\mathrm{iBALT}\;\mathrm{surfaces}}{\mathrm{Total}\;\mathrm{lungs}\;\mathrm{surfaces}}\right)X\;100$.

### Cell surface and intracellular staining

Mediastinal lymph node (MLNs) and lungs were harvested at specific time point (3/6/9 dpi). MLNs were collected and dissociated between microscope slides in FACS buffer (PBS/0.5% BSA/2 Mm EDTA) before the extracellular staining. For the lungs, they were collected in RPMI-1640 medium supplemented with recombinant Grade I DNAse I (10 U/ml, Merck) and collagenase A (1 mg/ml, Roche, Basel, Switzerland). Lung dissociation was done using the GentleMACS (Miltenyi Biotec; Bergisch Gladbach, Germany) performing the two specific programs with an incubation at 37°C for 20 min in between. To lyse erythrocytes, Ammonium-Chloride-Potassium lysing Buffer (ACK) was added to cell suspensions for 1 min. For extracellular staining, cells of interest were stained with a mix of antibodies diluted in FACS buffer at 4°C in the dark for 20 min. For IAV-specific T-cell responses, cells were monitored using pentamer staining (H-2 Db/SCLENFRAYV (PA_224–233_), Proimmune, Oxford, England). To exclude dead cells, fixable viability dye conjugated to iFluor860 maleimide (AAT Bioquest; California, USA) was used. To study the production of IFNγ by VM T cells, a mix of rIL-12/rIL-18 (10 ng/ml; MBL, Nagoya, Japan) was added to the cell culture for 4 h with Brefeldin A (1 µg/ml) (GolgiPlug^TM^; Bd Biosciences, California, United States) during the last 2 h before the extracellular staining and cells fixation with the BD Fixation/Permeabilization kit following the manufacturer protocol and the intracellular staining at room temperature in the dark for 30 min. To study the production of IFNγ by IAV-specific CD8 T cells, PA_224–233_ peptide (2 µM; Kaneka Eurogentec S.A., Seraing, Belgium) was added to the cell culture for 4 h with Brefeldin A (1 µg/ml) (GolgiPlug^TM^; Bd Biosciences, California, United States) before the extracellular staining, cells fixation, and intracellular staining. Finally, to study the Eomes expression in VM T cells, cells from the lungs were fixed and permeabilized using the Foxp3-Staining buffer kit (eBiosciences, Frankfurt, Germany) following the one-step manufacturer protocol. Antibodies used for flow cytometry are listed in the Supplementary materials Table S3. To get rid of other cells than cDC1s in the lungs and MLNs, we used a lineage (LIN) composed of the following markers: CD3, CD19 and NK1.1. Samples were acquired on CytoFLEX LX (6 lasers, Beckman Coulter) and analyzed using FlowJo Software (Tree Star, Inc).

### Statistical analysis

Data are expressed as mean +/ – SEM. Statistical comparison was made using GraphPad Prism 8 (GraphPad Software; San Diego, USA) and statistical test is specified in the legend of each Figure. Shapiro–Wilk test was used for normality test of the data and to choose the appropriate statistical test for normal distribution (One-way ANOVA (Holm-Sidak’s multiple comparisons test) or Two-way ANOVA (Sidak’s multiple comparisons test)) and non-normal distribution (Kruskal–Wallis test (Dunn’s multiple comparisons test)). *p* values less than or equal to 0,05 were considered significant.

## Results

### Maternal supplementation with L. rhamnosus or B. lactis decreases neonatal susceptibility to respiratory influenza virus infection

**Figure 1.**
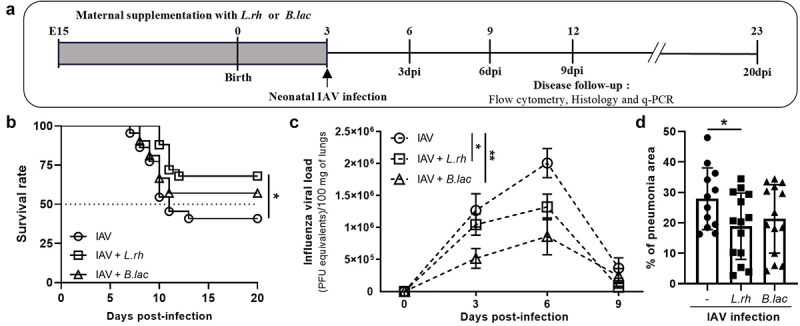
Maternal supplementation with *L.rh* or *B.lac* confers a better global protection against IAV infection, limits the viral load and the virus-induced damages in the lungs of neonates. (a) protocol used to study the neonatal immune responses against IAV (innate and adaptative). (b) Survival was observed for 20 dpi and plotted as percentages over time (mantel-cox test) (*n* = 21–25). (c) The viral load in the neonatal lungs was followed by q-PCR at 3/6/9 dpi (*n* = 6–15). statistical significance was determined by a two-way ANOVA (Tukey’s multiple comparisons test). (d) lung health was analysed by histology (H&E coloration) at 6 dpi.The percentage of pneumonia area was measured (*n* = 12–14). Statistical significance was determined by an unpaired t test between the IAV and IAV + probiotics (*L.rh* or *B.lac*). *p < 0.05 **p < 0.01 data from three different experiments.

To explore whether perinatal probiotic exposure affects the severity of viral respiratory infection in offspring, pregnant mice were daily supplemented with 2 × 10^8^ CFU of *L. rhamnosus (L.rh)* or *B. lactis (B.lac)* from embryonic day 15 to post-partum day 3. Pups born from probiotic-supplemented mothers exhibited no noticeable difference in general growth compared with control littermates as previously reported.^25^ To study the neonatal innate and adaptive immune responses, we followed the protocol illustrated in Figure 1a. First, 3-day-old neonates from untreated or probiotic-treated mothers were exposed to a sublethal dose (nLD50) of A/PR8 influenza virus (IAV) to evaluate their resistance for 20 d (Figure 1b). We observed a significant increase of the survival rate among pups born to *L.rh*-treated mothers compared to those from untreated mothers. The same tendency was observed with the *B.lac* supplementation. Then, we evaluated the viral burden in the offspring of untreated and probiotic-treated mothers (Figure 1c). Compared to neonates from untreated mothers, we observed that *L.rh* or *B.lac* treatment significantly reduces the viral loads throughout IAV infection. We then checked if this decreased viral load could be linked to a better lung health at the peak of the A/PR8 influenza virus infection (6dpi). To do so, histological sections of the infected lungs were performed. No edema, no hemorrhage was observed in the model. Severe, focal or diffuse alveolitis and interstitial inflammation were observed in collapsed lung area with focus of pneumonia in all groups (Supplementary materials Table S4). Interestingly, the percentage of pneumonia area was decreased in the *L.rh*-treated group compared with the infected untreated group (-) and a positive tendency was spotted for the *B.lac*-treated group (Figure 1d). All together, these observations indicate that maternal *L.rh* or *B.lac* exposure inhibits viral replication and limits primary IAV-induced damages at pulmonary barriers in neonates.

### The IAV-induced recruitment of cDC1s to the neonatal mediastinal lymph nodes and the subsequent CD8 T cell response are enhanced by the maternal supplementation with L. rhamnosus and B. lactis

**Figure 2.**
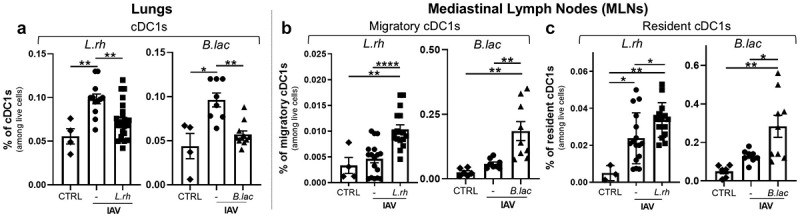
Maternal supplementation with *L.rh* or *B.lac* increases the migration of neonatal cDC1s from the lungs to the mediastinal lymph nodes (MLNs) following PR8 influenza infection. Three-day-old neonates from *L.rh*-treated, *B.lac*-treated or untreated (-) mothers were infected with IAV. Uninfected neonates from untreated mothers were used as controls (CTRL). The lungs and the MLNs were collected at 3 dpi. (a) The total cDC1s in the lungs, (b) the CD103-expressing migratory cDC1s, and (c) the CD8.

We focused on cells that bridge innate and adaptive responses during viral infections, specifically cDC1s (Figure 2). Three-day-old neonates from *L.rh*-treated, *B.lac*-treated or untreated mothers were infected with IAV. Their lungs and mediastinal lymph nodes (MLNs) were collected for cytometry at 3 dpi. We observed that maternal exposure to *L.rh* and *B.lac* reduced the percentage of cDC1s (CD45^+^MHCII^+^LIN^−^CD11c^+^CD26^+^XCR1^+^SIRPα^−^) in the IAV infected neonatal lungs (Figure 2a) while increasing the percentage of CD103-expressing migratory cDC1s (CD45^+^MHCII^+^LIN^−^CD11c^+^CD26^+^XCR1^+^CD103^+^) and CD8α-expressing resident cDC1s (CD45^+^MHCII^+^LIN^−^CD11c^+^CD26^+^XCR1^+^CD8^+^) in the MLNs (Figure 2b–c). While the observed reduction of cDC1s in the lungs, coupled with their increase in the mLNs, is consistent with migration, this alone does not definitively prove the movement of cells from the lungs to the mLNs. However, given the intranasal administration of IAV, the lungs are the most plausible source of CD103^+^ migratory cDC1s in the mLNs.

**Figure 3.**
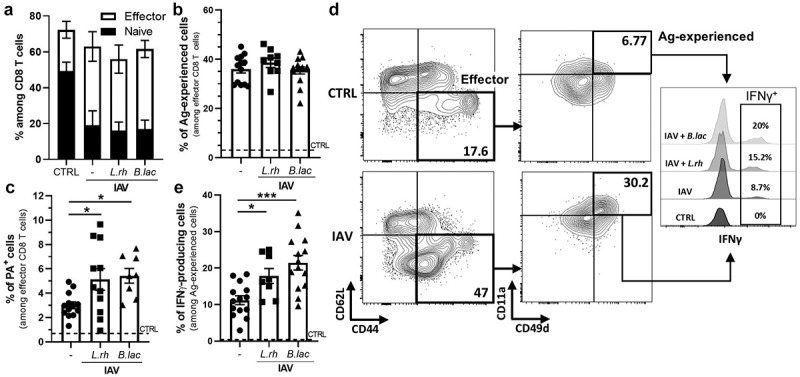
Maternal supplementation with *L.rh* or *B.lac* increases IAV-specific effector CD8 T cell responses. Three-day-old mice from *L.rh*-treated, *B.lac*-treated or untreated (-) mothers were infected with IAV (PR8-strain) and the lungs were harvested at 9 dpi to study the specific IAV CD8 T cells. Uninfected neonates from untreated mothers were used as controls (CTRL) and plotted as a dotted line. (a) The distribution of naïve and effector CD8 T cells and (B) the percentage of Ag-experienced effector cells were measured. (c) The percentage of PA-specific cells among the effector CD8 T cells was also determined. To study their capacity to produce IFN-γ, lung cells were stimulated for 4 hours with the IAV-specific peptide PA(224–233) and analyzed by flow cytometry. (d) The gating strategy and (e) the frequency of IFNγ-producing cells among Ag-experienced effector cells are shown. Statistical significance was determined by an ordinary one-way ANOVA (Dunnett’s comparisons test to untreated (-) group). *p < 0.05 ***p < 0.001. Data from two or three different experiments (*n* = 8–17/group).

Next, we tested if this phenomenon might lead to a stronger adaptive CD8 T cell response. Indeed, early during the infection, the migratory cDC1s are the main type of cells to prime naïve CD8 T cells in the MLNs while later the resident cDC1s play the main role.^43^ To test this, we harvested the lungs at 9dpi, the time required to establish an IAV-specific CD8 T cell response in neonates (Figure 3).^7^ As expected, IAV infection triggered a switch from naïve CD8 T cells (CD62L^+^CD44^−^) to effector CD8 T cells (CD62L^−^CD44^+^) in pups, with no significant effect of the mother supplementation (Figure 3a). We further deeply analyzed the effector CD8 T cell responses by investigating Ag-experienced effector cells identified by their up-regulation of both CD11a^44^ and CD49d^45^ integrins. These Ag-experienced effector cells appeared in all IAV infected neonates at 9 dpi, regardless of the mother treatment (Figure 3b).

To evaluate the impact of the maternal supplementation on effector CD8 T cells function and specificity, we used the polymerase acidic IAV-specific peptide (PA_(224–233)_) as the dominant epitope for the IAV responses in neonates^46^ either for the *in vitro* stimulation or for the identification of IAV-specific CD8 T cells. Using the PA-specific MHCI pentamer, we observed that both probiotic treatments significantly increased the frequency of PA-specific effector CD8 T cells at 9 dpi (Figure 3c). We then measured the production of IFN-γ, the main pro-inflammatory cytokine produced by CD8 T cells, as shown in the gating strategy (Figure 3d).

Interestingly, these Ag-experienced effector CD8 T cells produced more IFN-γ in infected neonates from both *L.rh* or *B.lac*-treated mothers compared with those from untreated mothers (Figure 3e).

Unexpectedly, in the same setting, we noticed that the CD4 T cell responses were also modified by the maternal probiotic supplementation. The percentages of IFN-γproducing effector CD4 T cells (CD45^+^CD3^+^CD4^+^CD8^−^CD62L^−^CD44^+^IFNγ^+^) were increased in the *L.rh-* and *B.lac*-treated groups compared with the untreated IAV group (Suplementary Figure S1A). Moreover, only the *L.rh*-treated group displayed a higher percentage of IL10-producing effector CD4 T cells (CD45^+^CD3^+^CD4^+^CD8^−^CD62L^−^CD44^+^IL-10^+^) compared with the untreated IAV group (Supplementary Figure S1B). It was reported that those effector CD4 T cells could help to create the perfect environment to clear the virus efficiently and to mount a better specific-IAV CD8 T memory response.^8,47^ We also observed that following *L.rh* maternal supplementation the production of IL-10 by neonatal CD8 T cells was greater compared to IAV infected pups (Supplementary Figure SC). This production of IL-10 during acute infection helps to avoid lethal pulmonary inflammation. In addition to flow cytometry, the up-regulation of *IL-10* was also observed by q-PCR in whole lungs of *L.rh* group compared to IAV group at 9dpi (Supplementary Figure S1D).

Taken together, these results showed that thanks to a potential better migration of the cDC1s to the MLNs, both *L.rh* and *B.lac* maternal exposures reinforce the IAV-specific effector CD8 T cell responses known to be less efficient in early life than in adulthood.^7,8^

### The virtual memory T cells are enhanced by the maternal supplementation with L. rhamnosus in the lungs of IAV infected neonates

**Figure 4.**
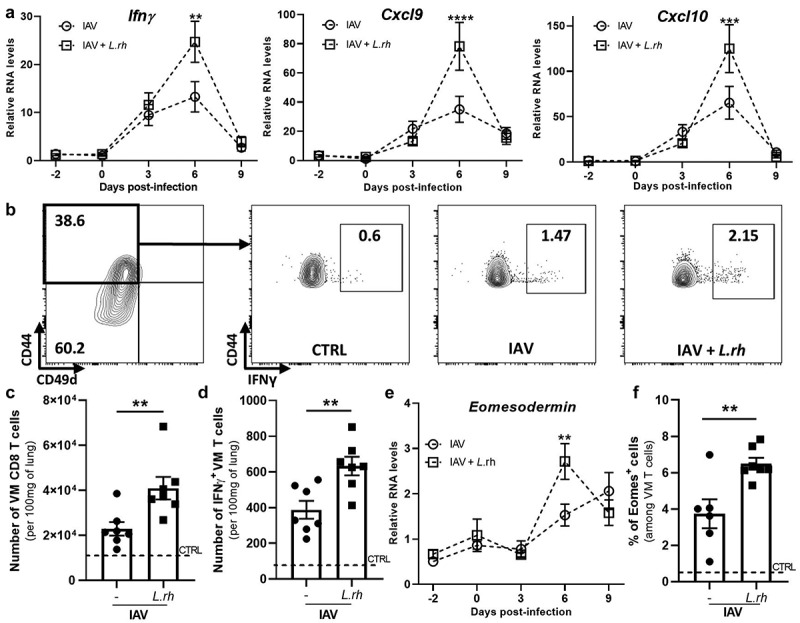
Maternal supplementation with *L.rh* increases the recruitment and activation of VM CD8 T cells. three-day-old mice from *L.rh*-treated or untreated mothers were infected with IAV (PR8-strain) and the lungs were harvested at 6 dpi to study the VM CD8 T cells by flow cytometry. Uninfected neonates from untreated mothers were used as controls (CTRL) and plotted as a dotted line. (a) The relative expression of Ifnγ, Cxcl9, Cxcl10 (e) and Eomesodermin were analyzed by RT-qPCR on the whole lungs. (*n* = 6–16) (b) the gating strategy used for the identification of IFN-γ–producing VM CD8 T cells by flow cytometry. (c–d) to study IFN-γ production, pulmonary cells were stimulated for 4 hours with r-IL12/r-IL18. (c) The number of VM CD8 T cells and (d) the number of IFN-γ-producing VM CD8 T cells normalized to 100 mg of neonatal lungs were determined. (f) The percentage of Eomes^+^ cells among VM T cells was measured by flow cytometry. Statistical significance was determined by an unpaired t test for the flow cytometry experiments and by a two-way ANOVA (Sidak’s multiple comparisons test) for the RT-qPCR. *p < 0.05 **p < 0.01 ***p < 0.001 ****p < 0.0001. Data from one representative experiment (out of 3).

We then investigated whether maternal probiotic exposure influenced other effector cells that might act earlier during the IAV infection, before the appearance of the effector CD8 T cells. To explore this, we first conducted a time-course analysis of the cytokines and chemokines mRNA synthesis in the lungs. We discovered that only IAV-infected neonates from *L.rh*-supplemented mothers displayed a higher expression of *Ifnγ*, *Cxcl9* and *Cxcl10* genes at 6dpi compared with the neonates from untreated mothers (Figure 4a and Figure S3). This observation led us to consider virtual memory (VM) CD8 T cells as potential contributor to elevated IFN-γ synthesis. Indeed, VM CD8 T cells are known to express the chemokine receptor CXCR3 to traffic toward CXCL9 and CXCL10 secretion site and to secrete rapidly IFN-γ via by-standard activation.^48,49^ It was reported that CD8 T cells have a greater rate of becoming VM T cells in neonates than in adults. The neonatal VM T cells would exhibit more rapid and innate-like functions than their adults’ counterparts that possess long-lived memory features.^50^ To investigate this hypothesis, we harvested lungs from infected neonates at 6dpi for cytometry analysis. We noticed that around 40% of CD8 T cells in the neonatal lungs were VM T cells, defined as CD8^+^CD44^+^CD49d^−^ (gating strategy: Figure 4b), as previously described.^51,52^ We observed that both the total number of VM T cells (Figure 4c) and the number of IFN-γ producing VM T cells (Figure 4d) were increased in IAV-infected neonates at 6dpi upon maternal *L.rh* supplementation. This increase was further supported by the elevated expression of *Eomesodermin* (*Eomes*), their main transcriptional factor. RT-qPCR confirmed the up-regulation of *Eomes* in the lungs of infected neonates (Figure 4e). In addition, we observed that the percentages of Eomes^+^ VM CD8 T cells were increased in IAV infected neonates from *L.rh*-treated mothers compared with untreated mothers (Figure 4f).

Further analysis revealed that most of these cells originated from the central memory T cell compartment (CD44^+^CD62L^+^) (Supplementary Figure S2A-B) as previously demonstrated in IAV infected adult lungs.^53^ As we highlighted an up-regulation of *Cxcl9* and *Cxcl10* by q-PCR, we checked and confirmed a higher CXCR3 receptor expression in those VM T cells to explain the higher recruitment of those cells in the *L.rh* group (Supplementary Figure S2C). Finally, gating into these CD62L^+^ VM T cells revealed an increased production of IFN-γ in the *L.rh*-treated group compared with the IAV group (Supplementary Figure S2D).

In contrast, the *B.lac* treatment elicited an earlier increase of *Ifnγ* expression at 3dpi in the lungs of IAV-infected pups compared with the untreated group (Supplementary Figure S3A). This observation could be linked with the higher decrease of viral loads observed at this time point in that probiotic-treated group (Figure 1c). As for the *L.rh*-treated group, we checked for the VM CD8 T cells but we did not observe an increase of those cells as well as their related-chemokines *Cxcl9* or *Cxcl10* at 3 dpi (Supplementary Figure S3A). In addition, there were no significant difference in the number of CD8 T cells, VM T cells and IFN-γ^+^ VM T cells, or the percentage of Eomes^+^ VM T cells between untreated and *B.lac*-treated groups (Supplementary Figure S3B-D).

Altogether, these results show that only the *L.rh* maternal supplementation positively impacted the recruitment of VM T cells into the neonatal lungs early during IAV infection. Upon neonatal infection, these cells are producers of IFN-γ, one of the main pro-inflammatory cytokines needed to fight IAV infection. However, the increased production of *Ifnγ* at 3dpi with the *B. lactis* supplementation was not attributed to VM T cells

### *The memory pool of CD8 T cells is increased in neonatally IAV-infected mice born to L. rhamnosus*- *or B. lactis-supplemented mothers*

**Figure 5.**
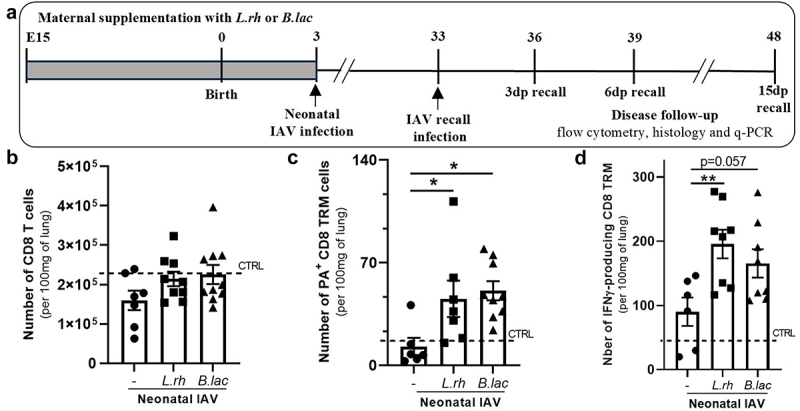
Maternal supplementation with *L.rh* or *B.lac* increases the pool of memory cells into the lungs of young mice 30 days after a neonatal IAV infection. (a) Experimental plan to study the memory response. Three-day-old mice from *L.rh*-treated, *B.lac*-treated or untreated (-) mothers were infected with IAV (PR8-strain) and grew until 30 days post-neonatal infection. Uninfected young mice (33-day-old) were used as controls (CTRL) and plotted as a dotted line. The lungs were harvested to study the pool of TRM cells. (b) The number of CD8 T cells and (c) the number of PA^+^ CD8 TRM cells were analyzed. (d) To study their capacity to produce IFN-γ, lung cells were stimulated for 4 hours with the PA(224–233) peptide. The number of IFN-γ^+^ CD8 TRM cells was determined by flow cytometry. Statistical significance was determined by an ordinary one-way ANOVA (Dunnett’s comparisons test to untreated (-) group). *p < 0.05 **p < 0.01.

Then, we examined the impact of maternal probiotic exposure on the induction of a virus-specific memory CD8 T cell response in the offspring. We decided to focus on one of the most important memory cell types: the tissue resident memory CD8 T cells (TRMs) as several studies in adult mice and humans have highlighted the importance of those cells in the protection against secondary infection with similar or heterologous virus.^54–56^ For this part of the study, we followed the protocol illustrated in Figure 5a, assessing the TRM population at 30 dpi. First, we observed a stable number of CD8 T cells in all young mice groups (Figure 5b) and no increased proportion of IAV-specific TRM (PA-MHCI pentamer^+^) in neonatally infected mice from untreated mothers compared to uninfected pups (Figure 5c) revealing a poor induction of memory CD8 T cells by the infection. Interestingly, maternal exposure to *L.rh* or *B.lac* significantly increased the pool of IAV-specific TRM in infected mice compared to maternal untreated pups. To study the effector capacity of those cells, we stimulated them *in vitro* with the PA peptide for 4 h and measured their production of IFN-γ by intracellular staining. The number of IFN-γ producing TRMs were significantly increased in the group of *L.rh group* and very closely in the *B.lac* group compared with infected (-) young mice from untreated mother (Figure 5d).

In conclusion, although maternal supplementation with *L.rh* or *B.lac* was administered prior to neonatal infection, notable effects were still evident in adulthood, beyond the acute phase of neonatal IAV infection. Specifically, we observed an expanded pool of TRM cells in these adults, exhibiting a more robust secondary response compared to infected young adults whose mothers had not received supplementation.

### Maternal supplementation with L. rhamnosus or B. lactis induces a protective memory response in the IAV-challenged young adult offspring

**Figure 6.**
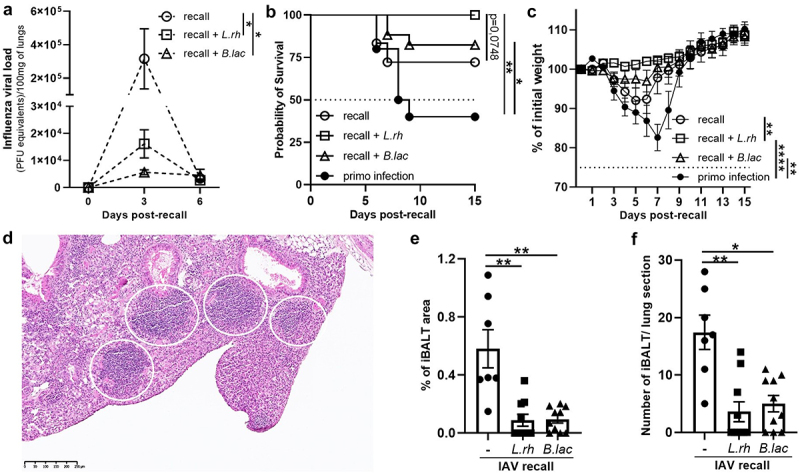
Maternal supplementation with *L.rh* or *B.lac* induces a protective memory responses against a secondary IAV challenge. Three-day-old mice from *L.rh*-treated, *B.lac*-treated or untreated (-) mothers were infected with IAV (PR8-strain) and grew until 30 d post-neonatal infection when a recall infection with a LD50 of the same IAV strain was done to follow (a) the viral load in the lungs at 3/6 d post-recall. (b) The survival and (c) the weight was followed for 2 weeks (Mantel–Cox test) (*n* = 10–18). (d) Histological staining was done at 5 dp recall and “induced bronchus-associated lymphoid tissues” (iBALT) were analyzed (white circle) (magnification x10). (e) The percentage of iBALT area and (f) the number of iBALT were measured (*n* = 7–10). Statistical significance was determined by a two-way ANOVA (Turkey’s multiple comparisons test) or a Kruskal–Wallis test (Dunn’s multiple comparisons test). *p < 0.05 **p < 0.01 ****p < 0.0001.

As we showed that maternal supplementation increased the pool of memory T cells in the lungs at 30 dpi, we next investigated whether these changes could enhance the protection of the young mice from a second IAV infection. To assess this, we analyzed the viral load, the survival rate, and the weight loss after a second sub-lethal dose of IAV 30 d after the primo infection (Figure 6).

We observed that, after challenge, both the *L.rh-* and *B.lac* maternal treatment significantly decreased the viral loads in the lungs compared with the untreated group (Figure 6a). We further noticed that in the IAV recall groups, compared with the mice born to untreated mothers, the *L.rh* treatment tends to increase the protection of young mice from death (*p* = 0,07) (Figure 6b). Weight monitoring revealed that the mice from the *L.rh* group maintained their body weight throughout the infection, contrasting with the weight loss observed in both IAV recall and the IAV primo infection groups (Figure 6c), a pattern consistent with the observed survival curve. On the other hand, mice from the *B.lac* group did experience some weight loss through the infection but less than IAV primo infection group, also matching the survival curve. Lung histology revealed no difference in the type of disease (pneumonia or broncho/pneumonia) (data not shown). However, the formation of ectopic lymphoid tissue called induced bronchus-associated lymphoid tissue (iBALT)^57^ (Figure 6d) was decreased (both in percentage area and in number) in the lungs of mice with the maternal supplementation with both probiotics (Figure 6e–f).

In summary, while young mice from probiotic supplemented mothers get infected a second time with IAV, they control better the viral replication with a reduced pulmonary iBALT compared with mice from the untreated mothers. Moreover, the *L.rh* supplementation appears to confer stronger protection as evidenced by a better body weight control and a trend to improve survival rate in the secondary infected mice.

### Maternal L. rhamnosus or B. lactis supplementation does not impact the global profile of intestinal microbiota composition

**Figure 7.**
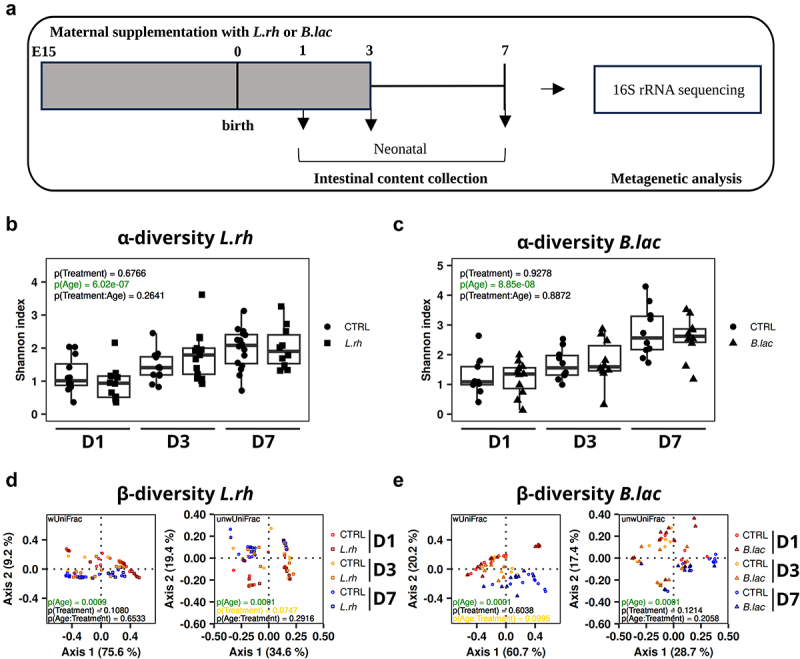
Evolution of the offspring’s gut microbiota α-diversity and β-diversity during the first days of life. (a) the protocol to study the gut microbiota composition in neonates. (b-c) the neonatal gut microbiota richness and evenness expressed as Shannon index for (b) *L.rh* and (c) *B.Lac* groups. Each pup group is depicted with ART ANOVA p-values for each factor (treatment, age) and their interaction (Treatment:age; significant values at *p* < 0.05 depicted in green text). (d–e) Principal coordinate analysis plots based on weighted (wUnifrac; left) and unweighted (unwUnifrac; right) UniFrac, respectively, are depicted with ADONIS p-values for each factor (age, treatment) and their interaction (Age:treatment) for (d) *L.rh* and (e) *B.lac* groups (significant values at *p* < 0.05 depicted in green, values at *p* < 0.1 are depicted in yellow. (*n* = 10–16, each litter contained 6–10 pups (D1), 5–8 pups (D3) or 3–4 pups (D7)).

Probiotic supplementation has the potential to influence the gut microbiota, the host metabolism and the immune system through various mechanisms. Therefore, we analyzed the gut microbiota composition of the mothers supplemented with *L.rh* or *B.lac*. None of the α- and β-diversity metrics differed with statistical significance between the control and treatment group (Supplementary Figure S4) suggesting that *L.rh* or *B.lac* supplementation does not impact the general profile of intestinal microbiota in the mothers. To better understand how maternal *L.rh* or *B.lac* supplementation impacts offspring immunity, we analyzed the composition of the gut microbiota in offspring from untreated, *L.rh* or *B.lac* supplemented mothers at different postnatal days. As expected, the gut microbiota richness and evenness increased during the first 7 d of development and independently of treatment (Figure 7b–c).^58^ Similarly, in the case of beta-diversity, the age was a significant (*p* = 0.0001) and a much stronger explanatory factor than treatment. Treatment only tended to be a significant factor (*p* = 0.0747, unwUniFrac) for *L.rh* compared to control, and none of the pairwise comparisons between treatments at any age were significant (Figure 7d–e). Even if maternal probiotic supplementation does not impact the global profile of gut microbiota composition, we cannot exclude changes in some specific bacterial taxa. To better characterize the effects of maternal probiotic treatment on the gut microbiota of the pups, a compositional data analysis was performed (Supplementary Figure S5). *L.rh* and *B.lac* supplementation in mothers affected differently the abundance of several taxa or ASV in the pups gut microbiota. The genus *Corynebacterium* was less abundant in pups from *B.lac*-treated mothers compared to control at D7, whereas unknown *Lactobacillaceae* and genera *Ligilactobacillus* and *Streptococcus* were less abundant in pups from *L.rh*-treated mothers at D1, and the latter also at D3 (Supplementary materials Table S5). Interestingly, *Streptococcus* ASV2 was present at higher relative frequencies in pups from treated mothers and, according to a BLAST comparison with *Streptococcus* RefSeq genomes, belonged to *Streptococcus acidominimus*, *Streptococcus azizii*, bacteria similar to them (a single-nucleotide difference from either). In contrast, ALDEx2 found no significant differences on either the ASV- or taxon-level. This indicated that maternal probiotic treatment does not drastically impact offspring gut microbiota composition, but slight effects on the pup microbiota are possible.

### *Maternal L. rhamnosus* or *B. lactis supplementation modulates neonate plasma metabolome*

**Figure 8.**
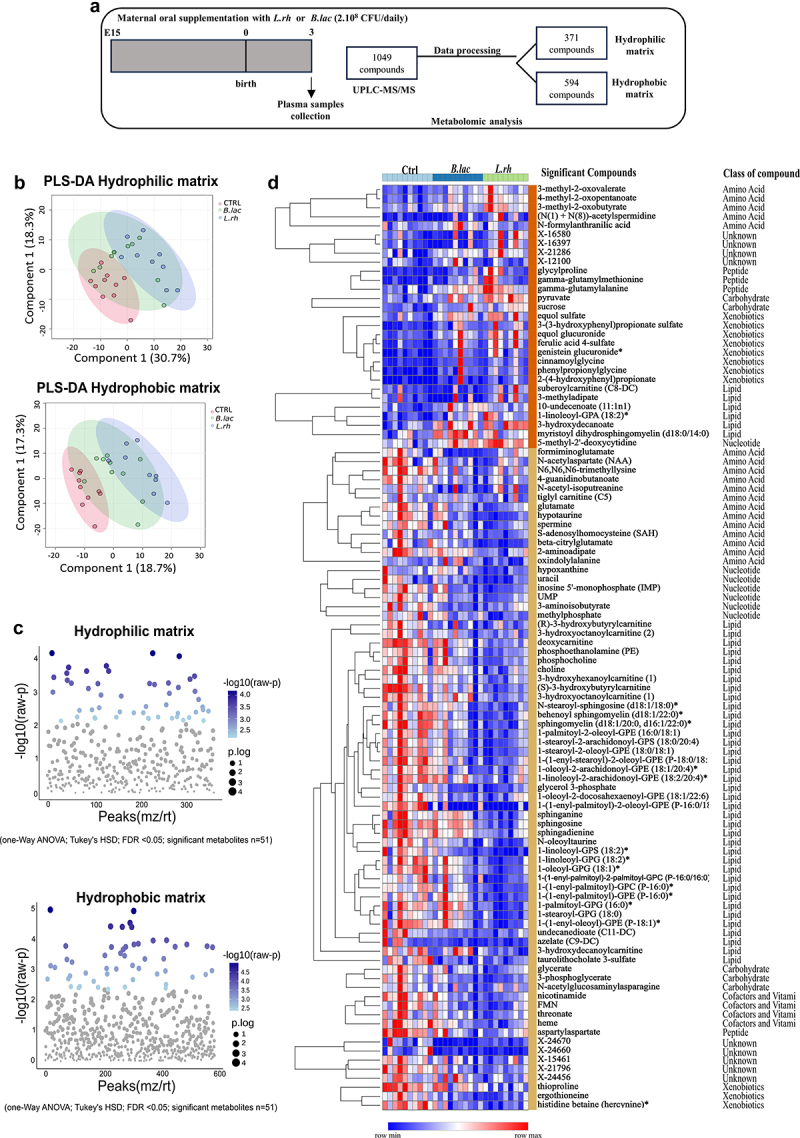
Maternal supplementation with *L.rh* or *B.lac* induces changes in the neonate plasma metabolome. (a) protocol to study the plasma metabolome of neonates. (b) Assessment of hydrophilic (panel up) and hydrophobic (panel down) plasma metabolites from neonates, by partial least squares discriminant analysis (PLS-DA). (c) One-way ANOVA (threshold FDR *p* < 0.05) with Tukey’s posttest in the both hydrophilic and hydrophobic matrix to identify metabolites with significant differences in the peak intensity comparing the pups from *B.lac* or *L.rh* treated-mothers versus control. (d) Hierarchical cluster analysis and heatmap of all significant metabolites selected by one-way analysis of variance (ANOVA) (*n* = 102 metabolites); *p*-value (FDR) <0,05 cutoff; post-hoc analysis by Tukey’s honestly significant difference (HSD) with raw *p*-value cutoff < 0,05. Each colored cell on the map corresponds to a normalized abundance of peak intensity value, with metabolites in rows and samples in columns. Just metabolites are hierarchical clustered by one minus Pearson correlation with linkage method by average, and grouped rows by cluster (K-means) and class of metabolites. *n* = 9–10. The figures were drawn via metaboanalyst software v 6.0 (https://www.Metaboanalyst.ca/) and phantasus v 1.21.5 (https://ctlab.Itmo.ru/phantasus).

The modulation of various cellular components of the immune system,^59^ including CD8 T cell activation and transition into memory cells^60^ has been associated with soluble microbial mediators. Therefore, we further investigated the impact of maternal supplementation with *L.rh* or *B.lac* on the offspring plasma metabolome. Our goal was to understand how maternal probiotic supplementation might prepare neonates to face infection. For this purpose, plasma samples were collected from 3-day-old pups for metabolomic study following maternal treatment from embryonic day 15 to post-partum day 3 (Figure 8a).

Using multivariate analysis with Partial Least Squares Discriminant Analysis (PLS-DA) model, we did not observe significant differences in the global composition of the metabolome, comparing the different experimental groups (CTRL, *L.rh* and *B.lac*), in both matrices of hydrophilic and hydrophobic compounds (permutation test for hydrophilic and hydrophobic data set p-value >0.05). However, there was a trend for some difference between *L.rh* and CTRL groups in the hydrophobic matrix. Specifically, longer distances are observed between the *L.rh* samples and the CTRL samples, whereas *B.lac* samples overlapped with the other two groups (Figure 8b).

We further performed univariate analysis by using one-way ANOVA (threshold FDR *p* < 0.05) with Tukey’s posttest on both hydrophilic and hydrophobic matrix to identify metabolites with significant differences in peak intensity comparing the pups from *L.rh* or *B.lac* treated-mothers versus control. The ANOVA analysis revealed 51 significantly altered metabolites by maternal probiotic supplementation from the hydrophilic set and 51 from the hydrophobic set with *p* value threshold 0.05 (Figure 8c).

To visualize these changes, we plotted all significant metabolites in a heatmap with hierarchical cluster analysis. Maternal treatment with *L.rh* and *B.lac* resulted in changes in various metabolite classes (amino acids, peptides, carbohydrates, nucleotides, cofactors and vitamins, xenobiotics, and unknown) in the plasma metabolome of pups, in comparison to neonates from untreated-mothers. The hierarchical organization showed two clusters: one (*n* = 29) with increased compound abundance by maternal probiotic supplementation, primarily xenobiotics, and another (*n* = 73) with reduced abundance by maternal probiotic supplementation, mainly composed of lipids (Figure 8d). The initial cluster exhibiting enhanced metabolites, primarily consisting of amino acids, lipids, and xenobiotics, appears to have influenced both treated groups. In the second cluster, decreased molecules, primarily lipids, appeared more pronounced in the group subjected to *L.rh* treatment. Pathway analysis based on enrichment procedures was performed using the list of significant metabolites. Interestingly, metabolites that were modified by maternal supplementation with *L.rh* and *B.lac* can be related to metabolic pathways, including: (1) Phospholipid biosynthesis; (2) Lipid metabolism; (3) Energy metabolism; (4) Amino acid metabolism; and (5) Other metabolic pathways (Supplementary materials Table S6).

These findings suggest that maternal supplementation with *L.rh* or *B.lac* not only increase substances related to microbial metabolism (xenobiotics)^39,61–63^ but also induced significant changes in the metabolic pathways of the offspring plasma metabolome.

### Maternal L. rhamnosus or B. lactis supplementation predominantly influences neonatal plasma metabolome changes through bacteria-derived metabolites

**Figure 9.**
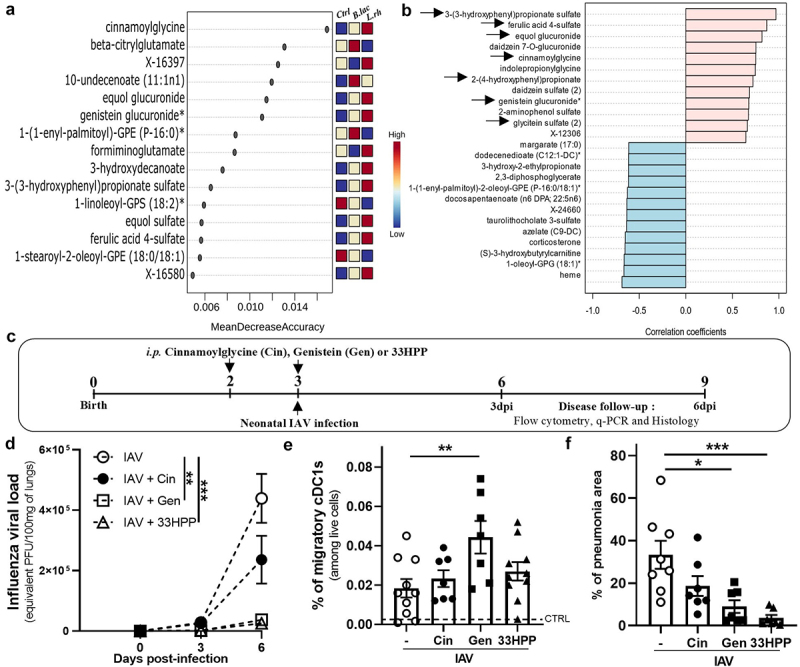
Identified xenobiotic metabolites (genistein and 3-(3-hydroxyphenyl)propionic acid) increase migratory cDC1s in MLN and/or viral load following PR8 influenza infection. (a) Rank of 15 most important metabolites from the 102 significant metabolites, identified by the random forest according to the mean decrease accuracy on the x-axis. Colored boxes indicate the relative peak intensity of the corresponding metabolite in each group. (b) Spearman rank correlation between 3-(3-hydroxyphenyl)propionate sulfate (33HPP-S) and metabolites. Group (ctrl, *B.lac* and *L.rh*) was selected as covariant of interest. The arrows point to significant metabolites from the xenobiotic class. (*n* = 9–10) (C) the protocol to study the impact of metabolites given to neonates during the influenza infection. (d) The viral load in the neonatal lungs was monitored by q-pcr at 3/6 dpi (*n* = 5–14). Statistical significance was determined by a two-way ANOVA (Tukey’s multiple comparisons test). Uninfected neonates from untreated mothers were used as controls (CTRL) and plotted as a dotted line. (e) The CD103-expressing migratory cDC1s were analyzed by flow cytometry in the MLN at 3dpi. Statistical significance was determined by an ordinary one-way ANOVA (Holm-Sidak’s multiple comparisons test). *p < 0.05 **p < 0.01 ***p < 0.001. Data are from two different experiments. (f) Lung health was analyzed by histology (H&E coloration) at 6 dpi and the percentage of pneumonia area was measured (*n* = 7–8). Statistical significance was determined by an unpaired T test or Mann–Whitney test between the IAV and IAV + metabolites (cin, Gen or 33HPP). *p < 0.05 **p < 0.01.

By using multivariate analysis with a random forest approach, we identified the top 15 most relevant variables from 102 significant metabolites considered as the most relevant for discriminating experimental groups. Among these, cinnamoylglycine emerged as the pivotal metabolite, important for group discrimination. Importantly, cinnamoylglycine has been described to be related to gut microbiota.^64^ Additionally, six other compounds (equol glucuronide, genistein glucuronide, 3-hydroxydecanoate, 3-(3-hydroxyphenyl) propionate sulfate, equol sulfate, and ferulic acid 4 sulfate) are also linked to the gut microbiota metabolism and were instrumental in differentiating the plasma from the control group than those from both treated groups. All these microbiota-related compounds were upregulated in the plasma of pups from mothers supplemented with *L.rh* or *B.lac* as shown in the color panel on the right of list (Figure 9a).

The main up- and down-regulated metabolites, considering the shared effect in both treatments (*L.rh* or *B.lac versus* CTRL), and only in the *L.rh* in the fold-change ratio are summarized in Supplementary materials Table S7. The most significant up-regulated metabolite was 3-(3-hydroxyphenyl)propionate (33HPP) sulfate. To assess the correlation of 33HPP with other microbiota-related metabolites, we conducted Spearman correlation across both hydrophilic and hydrophobic compound matrices. Interestingly, we identified a positive correlation between 33HPP-S and several other significant xenobiotics (highlighted with arrows) like cinnamoylglycine or one of the main phytoestrogen isoflavone genistein (Figure 9b). These findings reinforce the demonstration that maternal probiotic supplementation increases microbial metabolite levels in the offspring plasma. In order to determine whether maternal transmission or breastfeeding may contribute to the changes seen in the plasma metabolites of neonates, we conducted the same data treatment and processing from metabolomic analysis from maternal plasma samples and the milk collected from the neonates’ stomachs. We concentrated on the significant compounds present in newborn plasma (Figure 9a) to analyze the additional data sets. Nevertheless, the significant metabolites identified as important compounds in neonate plasma were not modified in milk and mother plasma (Supplementary Figure S6). The data indicate that the rise in xenobiotics in neonatal plasma does not probably result from direct maternal transfer or breastfeeding, but rather from more intricate pathways.

### Microbiota-derived metabolite supplementation protects neonates from influenza infection

To investigate the role of microbiota-derived metabolites in neonatal immune protection against IAV infection, neonates were treated with cinnamoylglycine, genistein, or 33HPP prior to IAV infection (Figure 9c). Compared to vehicle-treated neonates, we observed that genistein and 33HPP pretreatment significantly reduce the viral burden until the peak of infection but not the cinnamoylglycine (Figure 9d). Interestingly, only genistein-treated neonates displayed an increased percentage of migratory cDC1s (Figure 9e) after IAV infection. Concerning the health of the lungs in those mice, severe focal or diffuse alveolitis and interstitial inflammation were observed in collapsed lung area (Supplementary Table S8) but 33HPP and genistein treated neonates had a reduced percentage of pneumonia area compared to vehicle-treated ones (Figure 9f). These results indicate that genistein and 33HPP metabolites increased upon maternal supplementation with *L.rh* or *B.lac* contribute to protective features observed with the probiotics like the control of the viral load, the lung protection, and for genistein the early migration of cDC1s into the MLNs.

## Discussion

First exposure and composition of the microbiota are known to have a long-term impact on the developing immune system as germ-free (GF) or antibiotic-treated mice are more susceptible than untreated SPF mice to infections such as influenza.^65^ Evidence also suggests that maternal respiratory viral infections can negatively impact the maternal microbiota, leading to a weaker immune response against the same type of infections.^66^ With this study, we bring new insights into the understanding of a novel preventive microbial approach that reinforces immunity against respiratory viral infections using probiotics through the mother-offspring axis. In the particular case of respiratory viral infections, most of the previous work on probiotic protocols were designed to study their effects when given directly to hosts with poor attempts on very young age that represent a “high-risk” population facing influenza infection. Despite growing evidence of the health benefits of probiotics, the immune mechanisms by which they exert their effects are not fully understood. So far, they were mostly documented through their ability to differentially regulate the production of anti- and pro-inflammatory cytokines and the control on the balance between types of T cell responses such as Th1/Th2, Treg and Th17 responses.^67^ The studies reported so far in adult mice on the use of probiotics from the genus *Lactobacillus* and *Bifidobacterium* to demonstrate beneficial effects against IAV infection documented a better survival, the improvement of health status, the decrease of viral burden in the lungs and the early activation of pro-inflammatory cytokines.^17–20^ Little data shed light on immune cells implicated in the described protections. Notably, only one study in neonates has demonstrated that intranasal administration of *Lactobacillus rhamnosus GG* improves the survival rate upon IAV infection. This increased protection was also observed with a heat-killed probiotics and was mediated through the TLR4/MyD88 signaling pathway and an early type I IFNs production.^22^

Here, we documented a new possibility to improve the protection of neonates against IAV infection through maternal probiotic supplementation with *Lacticaseibacillus rhamnosus* VES001 (*L.rh*) or *Bifidobacterium animalis subsp. lactis* VES002 (*B.lac*) through slight different effector T cell activation. Indeed, compared to neonates from untreated mothers, pups from *L.rh*-treated mothers demonstrated higher survival rates with improved viral control and reduced tissue damages. These benefits were associated with increased IAV-specific CD8 T cells, elevated IFN-γ production, and early recruitment of virtual memory T cells. Maternal *L.rh* supplementation also boosted IAV-specific tissue-resident memory T cells and reduced iBALT formation, offering better protection against secondary infection and improved survival outcomes in adulthood. Although neonates from *B.lac*-treated mothers showed also lower viral loads, they displayed a slight survival improvement and a less pronounced tissue damage control. *B.lac* could increase IAV-specific CD8 T cells and IFN-γ production but not virtual memory CD8 T cells compared to *L.rh*. While *B.lac* also enhanced IFN-γ-producing TRM cells and reduced iBALT formation, it provided limited body weight maintenance and survival benefits during secondary infection. Overall, *L.rh* supplementation delivered more robust protection against both primary and secondary IAV infections compared to *B.lac*.

Among the cells that are crucial to eradicate IAV, the CD8 T cells are the main ones. To mount a robust CTL response, the naïve CD8 T cells need to be activated by the cDCs, especially by the cDC1s. Like *L.rh*, maternal *B.lac* supplementation reduced the percentage of cDC1s in the lungs while increasing CD103-expressing migratory cDC1s and CD8α-expressing resident cDC1s in the MLNs, supporting antigen processing and presentation. This potential improved migration could overcome the deficit of migratory CD103^+^ DCs to transport and to process antigens that was reported with another neonatal respiratory viral infection model.^68^ Moreover, we observed an increased frequency of resident cDC1s into MLNs potentially improving the development of efficient effector CD8 T cells by increasing the cross-presentation. Although the exact mechanisms are not fully understood, the transfer of Ag between migratory and resident cDC1s could occur directly (cell contact) or indirectly through the production of exosomes, soluble Ag or through the transfer of Ag-MHCI complexes.^69^ Concerning the CD8 T cell response in mice, two main markers, CD62L (L-selectin) and CD44 (Pgp-1), characterize the differentiation of naïve CD8 T cells into the effector and memory CD8 T cells following antigen presentation by the cDCs.^70,71^ Upon activation, effector CD8 T cells (CD44^+^CD62L^−^) migrate to the site of infection to mediate pathogen clearance and then undergo a contraction phase to build the memory pool.^72^ This stronger, specific, and faster primary CD8 T cell response observed in the probiotic treated groups is likely to contribute to the protective mechanisms upregulated by the maternal probiotic supplementation. In addition, we showed that beside producing IFN-γ, CD8 T cells produce IL-10 which help to balance the IAV response to avoid cytokine storm that could lead to lethal pulmonary inflammation and destruction.^73^ This was particularly the case for the infected *L.rh* group compared to the non-treated group and could help to explain the decreased pneumonia area observed in the lung of *L.rh*-treated group. Moreover, the environment to mount a better CD8 T cell memory response could be improved by the production of IFN-γ and IL-10 by activated CD4 T cells in both probiotic treated groups. Indeed, some studies have underlined the importance of CD4 T cells in the establishment of the appropriate environment for the CD8 T cells response (IFN-γ production) and future memory formation (IL-10 production).^74^

While adaptive responses are essential for eliminating IAV, early control of viral burden in the lungs may involve other immune cells.^75^ At 6 d post-IAV infection, we observed a strong increase of activated VM CD8 T cells in the lungs of the offspring from *L.rh* supplemented mother compared with untreated mother. The VM T cells come mainly from the central memory compartment and are better equipped with effector function (IFN-γ production). These observations of possible protective effects of VM T cells during neonatal IAV infection are the first ones even if we did not understand clearly the mechanisms involved. Indeed, VM T cells have been shown to arise during the first week of life and could be one of the lymphocytes responsible for the early protection of neonates.^51^ Moreover, those cells activate more rapidly, expand more quickly than naïve CD8 T cells in adults^76^ and form the main CD8 T cell-mediated immune response in aged mice.^77^ Concerning the *B.lac* supplementation, VM T cells were not the cells behind the increase of *IFNγ* at 3dpi. Other innate cells, like neutrophils that have been described during the early phase of infection, could be involved^78^ and further studies are needed to uncover them.

Multiple studies have established a link between a robust memory CD8 T cell pool after primary infection with some IAV strains and a better protection (survival, weight balance, virus loads, …) against a secondary infection with homologous and heterologous influenza viruses.^79–81^ We demonstrated for the first time that both maternal probiotic supplementations help to increase the specific TRM pool and to protect against a secondary infection. Accordingly, the microbiota’s importance in inducing memory antigen-specific CD8 T cells against early-life influenza infection was recently described.^82^ This improved protection upon reinfection in probiotic supplemented-groups may also be explained by a better production of virus-specific antibodies induced during the primary infection like demonstrated in other studies.^83^ Indeed, the size of virus-specific memory pool generated during primary infection is a good insight for the effectiveness of homologous but also heterologous immunity.^80^ Interestingly, during this recall IAV infection, we noticed the appearance of iBALT. Those structures have been highlighted in adult mice as common and important to fight a secondary infection when secondary lymphoid organs (SLOs) are lacking.^57,84^ In our study, those iBALTs were only observed after a recall infection in young adults having faced the primary infection in early life. This could be explained by a lack of SLOs or a poor induction of memory CD8 T cell pool in those infected neonates. Interestingly, young adults from both probiotics supplemented mothers, that are the only ones to display a better pool of memory CD8 T cells, did not have these iBALT. More studies are needed to reveal the role of those iBALT in early life respiratory infections.

To determine how maternal probiotic supplementation enhances neonatal IAV immune responses, we examined mother and pup intestinal microbiota. We found that both probiotics supplementation did not change the alpha or beta diversity of the maternal and offspring’s gut microbiota, although slight effects on the abundance of certain taxa in the pup gut microbiota remain possible. Moreover, we observed enrichment and a development of the gut microbiota over time organically in all pups, similarly to other studies.^58,85^ Chi et al., that directly supplementing newborns with *B.lac*, demonstrated that the effect of the probiotic on the microbiota composition was transient with an increase only in alpha diversity at 1 and 6 weeks after supplementation compared to newborns who did not receive the probiotic, but this effect disappeared after 12 weeks.^86^ Thus, our evidence suggests that mother probiotic supplementation is safe to keep the normal development of the offspring gut microbiota. Importantly, for some probiotics, despite no changes in gut microbial diversity is observed, effective effect on health have been shown.^87^ Indeed, probiotic supplementation may have an ecological impact since the host and gut bacteria’s metabolisms interact to create networks synthesizing metabolites that regulate both entities.^88,89^ Therefore, changes in microbial metabolism and metabolites may mediate immunoregulation.

Following our metabolomic analysis, Cinnamoylglycine emerged as a key compound in differentiating the plasma metabolome profile of the three experimental groups (Figure 9a). However, its treatment in neonates did not protect against IAV infection in early life and could thus not be responsible for the observed probiotic effects. Interestingly, the xenobiotic 3-(3-hydroxyphenyl) propionate sulfate (33HPP), that can be produced by colon bacteria through the metabolism of flavonoids (polyphenols), was the metabolite exhibiting the most significant increase in pup plasma from both supplemented groups (Supplementary materials Table 5).^90^ In our study, the origin of flavonoids or 33HPP is less clear and could derive from the polyphenols contained in whole grain cereals constituting mice diet.^91^ Importantly, 33HPP is associated with a robust ability to reduce serum lipid levels by upregulating the peroxisome proliferator-activated receptor alpha (PPARα) in hepatocytes.^92^ This property of 33HPP could be responsible for the drastic reduction of serum lipids observed in both probiotics-treated groups, with a more pronounced reduction of lipids in *L.rh* neonates that can be potentially in link with their higher 33HPP-S upregulation compared to *B.lac* mice (Figure 8d). Interestingly, other metabolites like Ferulic Acid and Genistein, that are upregulated in probiotics-treated groups with a higher increase in *L.rh*, can also increase oxidation of lipids.^93–96^

Remarkably, upon examining the metabolites positively correlated with 33HPP (Figure 9b) we observed that the majority – including genistein, daidzein, equol, and glycitein are isoflavonoid compounds that could have potential antiviral properties.^97–99^ Unlike 33HPP, which is derived from flavonoids through the metabolism of bacteria in the large intestine (C-ring cleavage of the flavonoid structure and dehydroxylation), these metabolites from isoflavonoids are generated by bacteria from the small intestine (via β-glucosidase).^100^ Among these, genistein or daidzein have already been linked to families of the probiotic bacteria used in this study (*Lacticaseibacillus* spp and *Bifidobacterium* spp) and are consistently significantly upregulated in *L.rh* and *B.lac* groups. Both *in vitro* and *in vivo* experiments showed the numerous ways of their antiviral action either on the host or the virus directly.^101^

We observed that the administration of 33HPP and genistein can control the IAV burden when administered directly to neonates. Additionally, genistein may indirectly contribute to protection against influenza infection in the offspring of probiotic-supplemented mothers via immune cells by increasing cDC1 in MLN. Those, respectively, flavonoid and isoflavonoid, can interact with cells expressing estrogen receptors (ER). ER activation may enhance the cytotoxic activity of CD8 T cells and increase the cytokine production by CD4 and CD8 T cells.^101^ Nevertheless, a direct effect of those metabolites on the virus itself cannot be totally excluded at this stage. Indeed, it has been shown that genistein can block the entry of IAV particles into the cells by interacting with the receptor of tyrosine kinases on lung epithelial cells^102^ and this metabolite can also inhibit the transcription step of viruses like African swine fever^103^ and Orf virus.^104^ Thus, it would be interesting to complete this study by measuring the IAV-specific CD8 T cells responses after metabolites administration and to measure the *in vitro* effect of 33HPP and Genistein on IAV replication.

By metabolomic analyses in mother plasma, milk and pup plasma, we found that the elevation of xenobiotics in neonatal plasma does not directly correlate with their concentrations in maternal plasma or milk. Complex mechanisms may play a role in sustaining elevated levels of compounds generated by the microbiota.^105^ This may relate to maternal probiotic supplementation’s capacity to regulate the fetal hepatic transcriptome.^106,107^ Moreover, it is possible that the neonate’s own microbiota, despite being young, is responsible for these modulations in levels of xenobiotics. Further analyses would be required to further delineate this relationship.

Our study presents some limitations. This is an exploratory study, where we used untargeted metabolomic and 16S analyses to generally assess the metabolic and compositional modulations of the gut microbiota induced by probiotic supplementation in order to unravel mechanisms involved in their protective effects against influenza virus infection. More precise techniques for absolute quantification of metabolites and total genome analysis of the intestinal microbiota could be complementary to deepen our understanding of the effects of probiotic supplementation on metabolome and gut microbiome. Analyzing metabolome profiles in neonates across other body compartments, including intestines, kidneys, urine, and intestinal luminal content would help interpret the metabolism and bioavailability of the key metabolites related to IAV protection.

In conclusion, we demonstrate for the first time that maternal probiotic supplementation enhances protection against IAV, likely through metabolites with immunomodulatory properties present in the offspring’s metabolome.

## Abbreviations

AM: alveolar macrophages
*B.lactis* (*B.lac*): *Bifidobacterium animalis subsp. lactis*
cDC1s: type 1 conventional dendritic cells
CTL: cytotoxic T lymphocyte
CTRL: control
dpi: days post-infection
Eomes: eomesodermin
FACS: Fluorescence Activated Cell Sorting
GF: germ-free
IAV: influenza A virus
iBALT: induced bronchus-associated lymphoid tissue
i.n: intranasal
*L.rhamonsus* (*L.rh*): *Lacticaseibacillus rhamnosus*
MLNs: mediastinal lymph nodes
RSV: respiratory syncytial virus
SCFAs: short chain fatty acids
TCR: T-cell receptor
TF: transcriptional factor
Th: T-helper
TRM: tissue resident memory
VM: virtual memory

## Supplementary Material

Supplemental Material

## Data Availability

The gut microbiota data used in this study have been deposited in the European Nucleotide Archive (ENA) at EMBL-EBI under the accession number PRJEB72811.
