# Supplementary material for: Maternal probiotic exposure enhances CD8 T cell protective neonatal immunity and modulates offspring metabolome to control influenza virus infection
Source: Gut Microbes. 2024 Dec 22;17(1):2442526. doi: 10.1080/19490976.2024.2442526 (PMC12931718; doi:10.1080/19490976.2024.2442526)
Supplement: Supplemental Material [file KGMI_A_2442526_SM7728.zip › kgmi-s-2024-1694-20241211215430/graphic/Supplementary material Tables.docx]

**Supplementary materials**

**Table 1:** List of primers used for real-time quantitative PCR experiments.

| **Gene** | **Forward primer** | **Reverse primer** | **Probe** |
| --- | --- | --- | --- |
| m-PPIA | 5’-TCC-TGG-CAT-CTT-GTC-CAT-GG-3’ | 5’-TTG-CCA-TCC-AGC-CAT-TCA-GT-3’ | 5’-TGC-TGG-ACC-AAA-CAC-AAA-CGG-CGG-TTC-CCA-3’ |
| m-HPRT | 5’-GGA-CCT-CTC-GAA-GTG-TTG-GAT-3’ | 5’-CCA-ACA-ACA-AAC-TTG-TCT-GGA-A-3’ | 5’-CAG-GCC-AGA-CTT-TGT-TGG-ATT-TGA-A-3’ |
| m-IFNγ | 5’-GGA-TGC-ATT-CAT-GAG-TAT-TGC-3’ | 5’-GCT-TCC-TGA-GGC-TGG-ATT-C-3’ | 5’-TTT-GAG-GTC-AAC-AAC-CCA-CAG-GTC-CA-3’ |
| m-Cxcl9 | 5’-GAA-CCC-TAG-TGA-TAA-GGA-ATG-CA-3’ | 5’-CTG-TTT-GAG-GTC-TTT-GAG-GGA-TT | 5’-CAT-CAG-CAC-CAG-CCG-AGG-CAC-G-3’ |
| m-Cxcl10 | 5’-GCC-GTC-ATT-TTC-TGC-CTC-AT-3’ | 5’-GCT-TCC-CTA-TGG-CCC-TCA-TT-3’ | 5’-TCT-CGC-AAG-GAC-GGT-CCG-CTG-3’ |
| m-Eomes | 5’-CCT-TCA-CCT-TCT-CAG-AGA-CAC-AGT-T-3’ | 5’-TCG-ATC-TTT-AGC-TGG-GTG-ATA-TCC-3’ | 5’-TCG-CTG-TGA-CGG-CCT-ACC-AAA-ACA-3’ |

**Table 2.** Significant metabolites identified via One-Way ANOVA in hydrophilic and hydrophobic data sets of offspring plasma

| **Class** | **Pathway** | **Metabolite** | **FDR** | **Tukey,s,HSD** | **Fold change (LAC/CTRL)** | **Fold change (LGG/CTRL)** |
| --- | --- | --- | --- | --- | --- | --- |
| Amino Acid | Histidine Metabolism | formiminoglutamate | 0,045 | LAC-CTRL | 0,45 | 0,73 |
|  | Guanidino and Acetamido Metabolism | 4-guanidinobutanoate | 0,012 | LAC-CTRL; LGG-CTRL | 0,79 | 0,70 |
|  | Lysine Metabolism | N6,N6,N6-trimethyllysine | 0,012 | LAC-CTRL; LGG-CTRL | 0,79 | 0,71 |
|  | Polyamine Metabolism | N-acetyl-isoputreanine | 0,045 | LAC-CTRL; LGG-CTRL | 0,69 | 0,77 |
|  | Leucine, Isoleucine and Valine Metabolism | 3-methyl-2-oxovalerate | 0,024 | LAC-CTRL; LGG-CTRL | 1,35 | 1,46 |
|  | Leucine, Isoleucine and Valine Metabolism | 4-methyl-2-oxopentanoate | 0,013 | LAC-CTRL; LGG-CTRL | 1,28 | 1,41 |
|  | Polyamine Metabolism | (N(1) + N(8))-acetylspermidine | 0,049 | LGG-CTRL | 2,02 | 3,51 |
|  | Alanine and Aspartate Metabolism | N-acetylaspartate (NAA) | 0,045 | LGG-CTRL | 0,80 | 0,72 |
|  | Glutamate Metabolism | glutamate | 0,038 | LGG-CTRL | 0,86 | 0,68 |
|  | Polyamine Metabolism | spermine | 0,045 | LGG-CTRL | 0,80 | 0,55 |
|  | Leucine, Isoleucine and Valine Metabolism | tiglylcarnitine (C5:1-DC) | 0,039 | LGG-CTRL; LGG-LAC | 0,92 | 0,78 |
|  | Lysine Metabolism | 2-aminoadipate | 0,012 | LGG-CTRL; LGG-LAC | 0,89 | 0,66 |
|  | Methionine, Cysteine, SAM and Taurine Metabolism | hypotaurine | 0,012 | LGG-CTRL; LGG-LAC | 0,79 | 0,54 |
|  | Glutamate Metabolism | beta-citrylglutamate | 0,009 | LGG-CTRL; LGG-LAC | 0,81 | 0,50 |
|  | Leucine, Isoleucine and Valine Metabolism | 3-methyl-2-oxobutyrate | 0,046 | LGG-CTRL | 1,18 | 1,30 |
|  | Methionine, Cysteine, SAM and Taurine Metabolism | S-adenosylhomocysteine (SAH) | 0,028 | LGG-CTRL | 0,80 | 0,55 |
|  | Tryptophan Metabolism | N-formylanthranilic acid | 0,045 | LGG-LAC | 1,24 | 0,96 |
|  | Tryptophan Metabolism | oxindolylalanine | 0,039 | LGG-LAC | 1,17 | 0,78 |
| Carbohydrate | Disaccharides and Oligosaccharides | sucrose | 0,038 | LAC-CTRL; LGG-CTRL | 1,62 | 1,68 |
|  | Aminosugar Metabolism | N-acetylglucosaminylasparagine | 0,035 | LAC-CTRL; LGG-CTRL | 0,82 | 0,76 |
|  | Glycolysis, Gluconeogenesis, and Pyruvate Metabolism | 3-phosphoglycerate | 0,010 | LAC-CTRL; LGG-CTRL LGG-LAC | 0,70 | 0,47 |
|  | Glycolysis, Gluconeogenesis, and Pyruvate Metabolism | glycerate | 0,045 | LGG-CTRL; LGG-LAC | 0,92 | 0,74 |
|  | Glycolysis, Gluconeogenesis, and Pyruvate Metabolism | pyruvate | 0,017 | LGG-CTRL | 1,32 | 1,65 |
| Cofactors and Vitamins | Nicotinate and Nicotinamide Metabolism | nicotinamide | 0,012 | LAC-CTRL; LGG-CTRL | 0,70 | 0,55 |
|  | Riboflavin Metabolism | FMN | 0,045 | LAC-CTRL; LGG-CTRL | 0,71 | 0,57 |
|  | Ascorbate and Aldarate Metabolism | threonate | 0,019 | LGG-CTRL; LGG-LAC | 0,85 | 0,69 |
|  | Hemoglobin and Porphyrin Metabolism | heme | 0,032 | LGG-CTRL | 0,90 | 0,76 |
| Lipid | Lysophospholipid | 1-linoleoyl-GPA (18:2)* | 0,039 | LAC-CTRL; LGG-CTRL | 1,76 | 2,16 |
|  | Fatty Acid Metabolism (Acyl Carnitine, Hydroxy) | (R)-3-hydroxybutyrylcarnitine | 0,012 | LAC-CTRL; LGG-CTRL | 0,80 | 0,70 |
|  | Glycerolipid Metabolism | glycerol 3-phosphate | 0,012 | LAC-CTRL; LGG-CTRL | 0,61 | 0,49 |
|  | Medium Chain Fatty Acid | 10-undecenoate (11:1n1) | 0,022 | LAC-CTRL; LGG-CTRL | 1,56 | 1,23 |
|  | Fatty Acid, Monohydroxy | 3-hydroxydecanoate | 0,045 | LAC-CTRL; LGG-CTRL | 1,28 | 1,25 |
|  | Sphingomyelins | behenoyl sphingomyelin (d18:1/22:0)* | 0,017 | LAC-CTRL; LGG-CTRL | 0,84 | 0,71 |
|  | Sphingomyelins | sphingomyelin (d18:1/20:0, d16:1/22:0)* | 0,006 | LAC-CTRL; LGG-CTRL | 0,84 | 0,65 |
|  | Plasmalogen | 1-(1-enyl-palmitoyl)-2-oleoyl-GPE (P-16:0/18:1)* | 0,006 | LAC-CTRL; LGG-CTRL | 0,83 | 0,31 |
|  | Plasmalogen | 1-(1-enyl-stearoyl)-2-oleoyl-GPE (P-18:0/18:1) | 0,011 | LAC-CTRL; LGG-CTRL | 0,76 | 0,61 |
|  | Phosphatidylethanolamine (PE) | 1-palmitoyl-2-oleoyl-GPE (16:0/18:1) | 0,006 | LAC-CTRL; LGG-CTRL | 0,73 | 0,55 |
|  | Phosphatidylethanolamine (PE) | 1-oleoyl-2-arachidonoyl-GPE (18:1/20:4)* | 0,019 | LAC-CTRL; LGG-CTRL | 0,70 | 0,59 |
|  | Phosphatidylethanolamine (PE) | 1-oleoyl-2-docosahexaenoyl-GPE (18:1/22:6)* | 0,046 | LAC-CTRL; LGG-CTRL | 0,66 | 0,59 |
|  | Phosphatidylserine (PS) | 1-stearoyl-2-arachidonoyl-GPS (18:0/20:4) | 0,006 | LAC-CTRL; LGG-CTRL | 0,64 | 0,40 |
|  | Endocannabinoid | N-oleoyltaurine | 0,007 | LAC-CTRL; LGG-CTRL | 0,63 | 0,46 |
|  | Fatty Acid, Dicarboxylate | undecanedioate (C11-DC) | 0,017 | LAC-CTRL; LGG-CTRL | 0,60 | 0,55 |
|  | Fatty Acid, Dicarboxylate | azelate (C9-DC) | 0,028 | LAC-CTRL; LGG-CTRL | 0,50 | 0,43 |
|  | Secondary Bile Acid Metabolism | taurolithocholate 3-sulfate | 0,049 | LAC-CTRL; LGG-CTRL | 0,43 | 0,36 |
|  | Phosphatidylethanolamine (PE) | 1-stearoyl-2-oleoyl-GPE (18:0/18:1) | 0,004 | LAC-CTRL; LGG-CTRL LGG-LAC | 0,75 | 0,51 |
|  | Lysophospholipid | 1-oleoyl-GPG (18:1)* | 0,003 | LAC-CTRL; LGG-CTRL LGG-LAC | 0,71 | 0,41 |
|  | Dihydrosphingomyelins | myristoyl dihydrosphingomyelin (d18:0/14:0)* | 0,022 | LAC-CTRL | 1,47 | 1,10 |
|  | Fatty Acid Metabolism (Acyl Carnitine, Hydroxy) | 3-hydroxyoctanoylcarnitine (2) | 0,016 | LGG-CTRL | 0,81 | 0,67 |
|  | Phospholipid Metabolism | choline phosphate | 0,050 | LGG-CTRL | 0,87 | 0,63 |
|  | Phospholipid Metabolism | choline | 0,018 | LGG-CTRL; LGG-LAC | 0,92 | 0,72 |
|  | Fatty Acid Metabolism (Acyl Carnitine, Hydroxy) | 3-hydroxyoctanoylcarnitine (1) | 0,012 | LGG-CTRL; LGG-LAC | 0,92 | 0,71 |
|  | Carnitine Metabolism | deoxycarnitine | 0,035 | LGG-CTRL; LGG-LAC | 0,89 | 0,70 |
|  | Fatty Acid Metabolism (Acyl Carnitine, Hydroxy) | 3-hydroxyhexanoylcarnitine (1) | 0,012 | LGG-CTRL; LGG-LAC | 0,88 | 0,69 |
|  | Phospholipid Metabolism | phosphoethanolamine | 0,045 | LGG-CTRL; LGG-LAC | 0,91 | 0,66 |
|  | Fatty Acid Metabolism (Acyl Carnitine, Hydroxy) | (S)-3-hydroxybutyrylcarnitine | 0,010 | LGG-CTRL; LGG-LAC | 0,81 | 0,62 |
|  | Fatty Acid Metabolism (Acyl Carnitine, Hydroxy) | 3-hydroxydecanoylcarnitine | 0,026 | LGG-CTRL; LGG-LAC | 0,96 | 0,74 |
|  | Lysoplasmalogen | 1-(1-enyl-palmitoyl)-GPC (P-16:0)* | 0,009 | LGG-CTRL; LGG-LAC | 0,91 | 0,65 |
|  | Ceramides | N-stearoyl-sphingosine (d18:1/18:0)* | 0,011 | LGG-CTRL; LGG-LAC | 0,86 | 0,65 |
|  | Sphingolipid Synthesis | sphinganine | 0,016 | LGG-CTRL; LGG-LAC | 0,92 | 0,62 |
|  | Lysoplasmalogen | 1-(1-enyl-palmitoyl)-GPE (P-16:0)* | 0,004 | LGG-CTRL; LGG-LAC | 1,00 | 0,60 |
|  | Plasmalogen | 1-(1-enyl-palmitoyl)-2-palmitoyl-GPC (P-16:0/16:0)* | 0,011 | LGG-CTRL; LGG-LAC | 0,84 | 0,56 |
|  | Sphingosines | sphingosine | 0,003 | LGG-CTRL; LGG-LAC | 0,88 | 0,56 |
|  | Sphingolipid Synthesis | sphingadienine | 0,006 | LGG-CTRL; LGG-LAC | 0,86 | 0,53 |
|  | Lysophospholipid | 1-stearoyl-GPG (18:0) | 0,045 | LGG-CTRL; LGG-LAC | 0,89 | 0,50 |
|  | Lysoplasmalogen | 1-(1-enyl-oleoyl)-GPE (P-18:1)* | 0,004 | LGG-CTRL; LGG-LAC | 0,76 | 0,44 |
|  | Lysophospholipid | 1-linoleoyl-GPG (18:2)* | 0,006 | LGG-CTRL; LGG-LAC | 0,73 | 0,39 |
|  | Lysophospholipid | 1-palmitoyl-GPG (16:0)* | 0,006 | LGG-CTRL; LGG-LAC | 0,88 | 0,35 |
|  | Lysophospholipid | 1-linoleoyl-GPS (18:2)* | 0,004 | LGG-CTRL; LGG-LAC | 0,51 | 0,23 |
|  | Phosphatidylethanolamine (PE) | 1-linoleoyl-2-arachidonoyl-GPE (18:2/20:4)* | 0,039 | LGG-CTRL | 0,87 | 0,61 |
|  | Fatty Acid Metabolism (Acyl Carnitine, Dicarboxylate) | suberoylcarnitine (C8-DC) | 0,045 | LGG-LAC | 0,84 | 1,28 |
|  | Fatty Acid, Dicarboxylate | 3-methyladipate | 0,045 | LGG-LAC | 2,08 | 1,26 |
| Nucleotide | Pyrimidine Metabolism, Cytidine containing | 5-methyl-2'-deoxycytidine | 0,012 | LAC-CTRL; LGG-CTRL | 1,46 | 1,57 |
|  | Pyrimidine Metabolism, Thymine containing | 3-aminoisobutyrate | 0,012 | LAC-CTRL; LGG-CTRL | 0,53 | 0,61 |
|  | Purine Metabolism, (Hypo)Xanthine/Inosine containing | inosine 5'-monophosphate (IMP) | 0,012 | LGG-CTRL | 0,67 | 0,36 |
|  | Pyrimidine Metabolism, Uracil containing | uridine 5'-monophosphate (UMP) | 0,014 | LGG-CTRL; LGG-LAC | 0,81 | 0,63 |
|  | Purine and Pyrimidine Metabolism | methylphosphate | 0,045 | LGG-CTRL; LGG-LAC | 0,88 | 0,61 |
|  | Pyrimidine Metabolism, Uracil containing | uracil | 0,012 | LGG-CTRL; LGG-LAC | 0,82 | 0,58 |
|  | Purine Metabolism, (Hypo)Xanthine/Inosine containing | hypoxanthine | 0,016 | LGG-CTRL; LGG-LAC | 1,01 | 0,37 |
| Peptide | Gamma-glutamyl Amino Acid | gamma-glutamylmethionine | 0,039 | LAC-CTRL; LGG-CTRL | 2,07 | 2,99 |
|  | Gamma-glutamyl Amino Acid | gamma-glutamylalanine | 0,012 | LAC-CTRL; LGG-CTRL | 1,62 | 1,75 |
|  | Dipeptide | aspartylaspartate | 0,032 | LAC-CTRL; LGG-CTRL | 0,70 | 0,55 |
|  | Dipeptide | glycylproline | 0,039 | LGG-CTRL | 2,21 | 3,69 |
| Untargeted | Unkow | X-21286 | 0,022 | LAC-CTRL; LGG-CTRL | 1,03 | 1,00 |
|  | Unkow | X-21796 | 0,006 | LAC-CTRL; LGG-CTRL | 0,74 | 0,59 |
|  | Unkow | X-24660 | 0,019 | LAC-CTRL; LGG-CTRL | 0,31 | 0,18 |
|  | Unkow | X-24670 | 0,006 | LAC-CTRL; LGG-LAC | 0,27 | 0,41 |
|  | Unkow | X-15461 | 0,035 | LGG-CTRL | 0,87 | 0,74 |
|  | Unkow | X-16397 | 0,038 | LGG-CTRL; LGG-LAC | 0,98 | 1,69 |
|  | Unkow | X-16580 | 0,050 | LGG-CTRL; LGG-LAC | 0,96 | 1,39 |
|  | Unkow | X-24456 | 0,039 | LGG-CTRL; LGG-LAC | 0,97 | 0,59 |
|  | Unkow | X-12100 | 0,023 | LGG-LAC | 1,20 | 0,88 |
| Xenobiotics | Food Component/Plant | ergothioneine | 0,010 | LAC-CTRL; LGG-CTRL | 0,64 | 0,54 |
|  | Benzoate Metabolism | 3-(3-hydroxyphenyl)propionate sulfate | 0,029 | LAC-CTRL; LGG-CTRL | 3,72 | 4,60 |
|  | Food Component/Plant | cinnamoylglycine | 0,017 | LAC-CTRL; LGG-CTRL | 2,81 | 2,55 |
|  | Benzoate Metabolism | phenylpropionylglycine | 0,009 | LAC-CTRL; LGG-CTRL | 2,38 | 2,01 |
|  | Food Component/Plant | equol glucuronide | 0,006 | LAC-CTRL; LGG-CTRL | 2,29 | 2,65 |
|  | Food Component/Plant | ferulic acid 4-sulfate | 0,016 | LAC-CTRL; LGG-CTRL | 2,21 | 2,82 |
|  | Benzoate Metabolism | 2-(4-hydroxyphenyl)propionate | 0,022 | LAC-CTRL; LGG-CTRL | 2,02 | 1,37 |
|  | Food Component/Plant | equol sulfate | 0,045 | LAC-CTRL; LGG-CTRL | 1,55 | 1,54 |
|  | Food Component/Plant | genistein glucuronide* | 0,006 | LAC-CTRL; LGG-CTRL | 1,53 | 1,91 |
|  | Food Component/Plant | histidine betaine (hercynine)* | 0,017 | LGG-CTRL | 0,54 | 0,31 |
|  | Chemical | thioproline | 0,014 | LGG-CTRL; LGG-LAC | 0,82 | 0,51 |

**Table 3:** List of antibodies used for flow cytometry experiments.


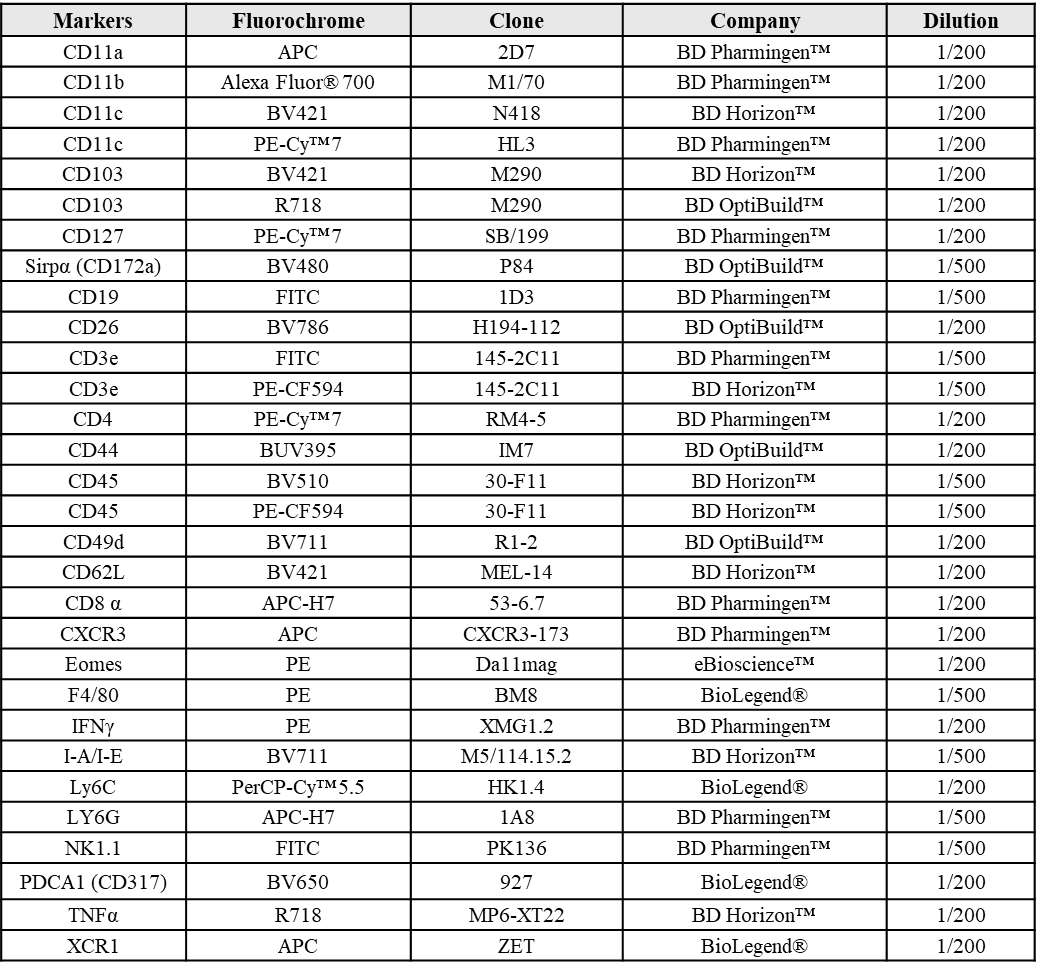


**Table 4** : Histology scoring


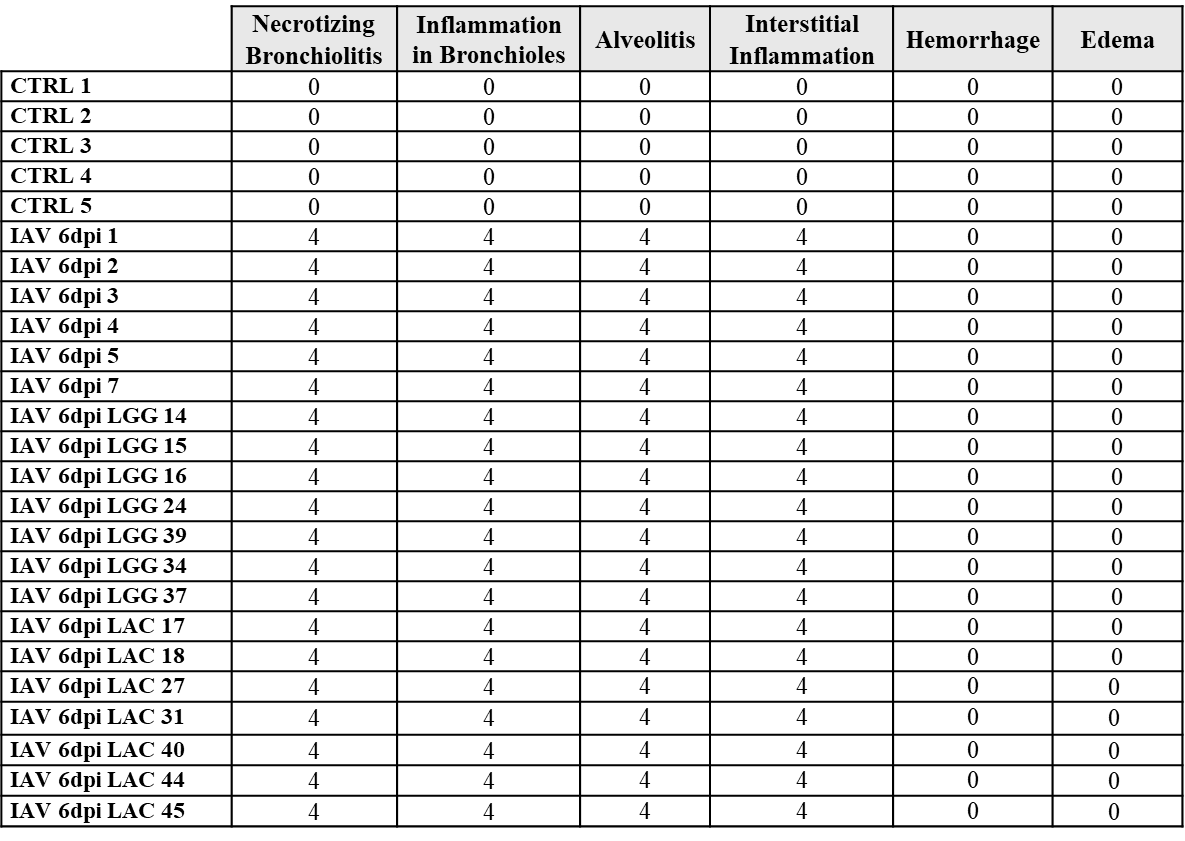


The severity of damage was scored on a scale ranging from **0** to **4**: **0** for none or very minor, **1** for mild, **2** for intermediate, **3** for moderately severe, and **4** for severe and widespread. CTRL = non-infected neonates.

**Table 5** : Several gut microbiota amplicon sequence variants (ASVs) and taxa were differentially abundant with statistical significance in pups from treated (*B.lac* or *L.rh*) or untreated (CTRL) mothers, based on ASV- and taxon-level analyses with ANCOM-BC.

| ***B.lac*** | **ASV** | **Age** | **Mean relative frequency  in CTRL [%]** | **Mean relative frequency  in *B.lac* [%]** | **ANCOM-BC  q-value** |
| --- | --- | --- | --- | --- | --- |
|  | *Streptococcus* ASV2 | D1 | 0.79 | 22.50 | 0,0010 |
|  | *Streptococcus* ASV5 | D1 | 1.09 | 0.15 | 0.0250 |
|  | *Streptococcus* ASV8 | D1 | 0.72 | 0.11 | 0.0250 |
|  | *Streptococcus* ASV15 | D1 | 0.16 | 0.04 | 0.0250 |
|  | *Streptococcus* ASV18 | D1 | 0 | 0.16 | 0.0250 |
|  | *Streptococcus* ASV23 | D1 | 0 | 0.12 | 0.0250 |
|  | **Taxon** |  |  |  |  |
|  | *Corynebacterium* | D7 | 0.92 | 0.09 | 0.0425 |
| ***L.rh*** | **ASV** | **Age** | **Mean relative frequency  in CTRL [%]** | **Mean relative frequency  in *L.rh* [%]** | **ANCOM-BC  q-value** |
|  | *Corynebacterium* ASV3 | D1 | 0.01 | 0.06 | 0.0149 |
|  | *Streptococcus* ASV2 | D1 | 5.75 | 9.83 | 0,0224 |
|  | unknown *Pasteurellaceae* ASV1 | D1 | 0.01 | 0.04 | 0.0149 |
|  | *Streptococcus* ASV12 | D3 | 0.14 | 0 | 0.0470 |
|  | *Streptococcus* ASV1 | D7 | 5.31 | 2.13 | 0.0474 |
|  | *Streptococcus* ASV2 | D7 | 0.18 | 2.59 | 0.0269 |
|  | **Taxon** |  |  |  |  |
|  | unknown *Lactobacillaceae* | D1 | 0.0490 | 0 | 0.0003 |
|  | *Ligilactobacillus* | D1 | 5.1720 | 1.69 | 0.0152 |
|  | *Streptococcus* | D1 | 39.52 | 27.59 | 0.0048 |
|  | *Streptococcus* | D3 | 35.74 | 18.48 | 0.0209 |

**Table 6:** Pathway analysis of the significant metabolites of offspring plasma

| **Pathway Name** | **Match Status** | **p** | **-log(p)** | **Holm p** | **FDR** | **Impact** |
| --- | --- | --- | --- | --- | --- | --- |
| Phosphatidylcholine Biosynthesis (1) | Phosphorylcholine, S-Adenosylhomocysteine, O-Phosphoethanolamine, L-1-phosphatidylserine | 0.0016549 | 2.7812 | 0.16384 | 0.16384 | 0.39024 |
| Sphingolipid Metabolism (5) | Sphinganine, O-Phosphoethanolamine; Ceramide (d18:1/18:0); Sphingosine, Phosphorylcholine | 0.0037677 | 2.4239 | 0.36924 | 0.1865 | 0.39611 |
| Carnitine Synthesis (5) | N6, N6,N6-Trimethyl-L-lysine; S-Adenosylhomocysteine; 4-Trimethylammoniobutanoic acid | 0.011222 | 1.9499 | 1.0 | 0.37033 | 0.38824 |
| Phospholipid Biosynthesis (1) | Glycerol 3-phosphate, Choline, Phosphorylcholine | 0.03821 | 1.4178 | 1.0 | 0.9457 | 0.1794 |
| Phosphatidylethanolamine Biosynthesis (1) | an L-1-phosphatidylethanolamine; , choline | 0.057735 | 1.2386 | 1.0 | 1.0 | 0.5 |
| Valine, Leucine and Isoleucine Degradation (4) | Alpha-ketoisovaleric acid; Ketoleucine, 3-Methyl-2-oxovaleric acid; S)-b-aminoisobutyric acid | 0.066286 | 1.1786 | 1.0 | 1.0 | 0.027012 |
| Betaine Metabolism (4) | S-Adenosylhomocysteine; choline | 0.10302 | 0.98706 | 1.0 | 1.0 | 0.1682 |
| Glycolysis (3) | 3-Phosphoglyceric acid; Pyruvic acid | 0.12322 | 0.90932 | 1.0 | 1.0 | 0.093021 |
| Glycerolipid Metabolism (2) | 3-Phosphoglyceric acid; Glycerol 3-phosphate | 0.15513 | 0.80931 | 1.0 | 1.0 | 0.22587 |
| Starch and Sucrose Metabolism (5) | Sucrose, 3-Phosphoglyceric acid; | 0.18848 | 0.72474 | 1.0 | 1.0 | 0.0 |
| Pyruvaldehyde Degradation (3) | Pyruvic acid | 0.19676 | 0.70607 | 1.0 | 1.0 | 0.0 |
| Glycerol Phosphate Shuttle (2) | Glycerol 3-phosphate | 0.22161 | 0.65441 | 1.0 | 1.0 | 0.33333 |
| Gluconeogenesis (3) | 3-Phosphoglyceric acid; Pyruvic acid | 0.23434 | 0.63015 | 1.0 | 1.0 | 0.1116 |
| Taurine and Hypotaurine Metabolism (4) | 2-aminoethanethiol dioxygenase | 0.24572 | 0.60957 | 1.0 | 1.0 | 0.0 |
| Glucose-Alanine Cycle (3) | Pyruvic acid | 0.24572 | 0.60957 | 1.0 | 1.0 | 0.1875 |
| De Novo Triacylglycerol Biosynthesis (2) | Glycerol 3-phosphate | 0.24572 | 0.60957 | 1.0 | 1.0 | 0.2 |
| Nicotinate and Nicotinamide Metabolism (5) | Niacinamide; S-Adenosylhomocysteine | 0.2576 | 0.58906 | 1.0 | 1.0 | 0.0 |
| Aspartate Metabolism (4) | N-Acetyl-L-aspartic acid; Inosinic acid | 0.28093 | 0.5514 | 1.0 | 1.0 | 0.0 |
| Cardiolipin Biosynthesis (1) | Glycerol 3-phosphate | 0.29178 | 0.53495 | 1.0 | 1.0 | 0.2987 |
| Histidine Metabolism (4) | S-Adenosylhomocysteine; Formiminoglutamic acid | 0.29259 | 0.53373 | 1.0 | 1.0 | 0.0090498 |
| (1) Phospholipid biosynthesis pathways; (2) Lipid metabolism Pathways; (3) Energy metabolism pathways; (4) Amino acid metabolism pathways; (5) Other metabolic pathways. | | | | | |  |

**Table 7:** Top significantly up or downregulated metabolites in offspring plasma metabolome born to mothers supplemented with probiotics, and possible links with antiviral protection.

| **Shared effects between *B.lac* or *L.rh*** | **Metabolite** | **Potential immunological connection** | **Ratio (*B.lac*:Ctl)** | **Ratio (*L.rh*:Ctl)** |
| --- | --- | --- | --- | --- |
|  | 3-(3-hydroxyphenyl)propionate sulfate | It is associated with a robust ability to reduce serum lipid levels^1^. | 3,72 | 4,60 |
|  | ferulic acid 4-sulfate | Compounds derivatives of ferulic acid exhibiting intense inhibitory activity during the early stage of flu infection^2^. | 2,21 | 2,82 |
|  | equol glucuronide | Isoflavones can bind estrogen receptor beta (ER). ERα signaling in conventional DCs can promote IFN-α and IL-6^3,4^. | 2,29 | 2,65 |
|  | cinnamoylglycine | It is related with PPAR activity and induce effector function of CD8+ T cells^5^. | 2,81 | 2,55 |
|  | genistein glucuronide* | Isoflavonoid compounds that could have potential antiviral properties^6^. | 1,53 | 1,91 |
|  | taurolithocholate 3-sulfate | It was associated with diminished COVID-19 vaccine-induced antibody responses in immunosuppressed inflammatory bowel disease patients^7^. | 0,43 | 0,36 |
|  | undecanedioate (C11-DC) | GPR84 agonist directly affects immune function and metabolic dysregulation, mediating IL-12 release^8^. | 0,60 | 0,55 |
| ***L.rh* effects** | (N(1) + N(8))-acetylspermidine | Induces dendritic cells tolerance by enhancing IL-10 production^9^. | n.s. | 3,51 |
|  | pyruvate | Is inhibited to entry into mitochondria, when TCR signaling activates PDHK1, promoting its conversion to acetyl CoA, and inducing metabolic changes in CD8+ T cells^10^. | n.s. | 1,65 |
|  | 3-methyl-2-oxobutyrate | Indirectly link with immune system regulation by energy metabolism via catabolism of branched-chain amino acids^11^. | n.s. | 1,30 |
|  | histidine betaine (hercynine)* | Related to CD8+ suppression^12^. | n.s. | 0,31 |
|  | inosine 5'-monophosphate (IMP) | The use of blockers of the enzyme that catalyzes IMP has been associated with improved response to anticancer immunotherapy^13^. | n.s. | 0,36 |
|  | spermine | Its reduction may be related to the production of N-acetylspermidine ^14^. | n.s. | 0,55 |
|  | S-adenosylhomocysteine (SAH) | Is is converted to homocysteine and adenosine in liver and is associate with interleukin-10 and interleukin-6 regulation^15^. | n.s. | 0,55 |

*Indicates a compound that has not been confirmed based on a standard, but we are confident in its identity (during MS/MS identification); n.s= non-significant.

**References Table 7**

1. Guo, J. *et al.* Protective Effects of Hydroxyphenyl Propionic Acids on Lipid Metabolism and Gut Microbiota in Mice Fed a High-Fat Diet. *Nutrients* **15**, 1043 (2023).

2. Antonopoulou, I., Sapountzaki, E., Rova, U. & Christakopoulos, P. Ferulic Acid From Plant Biomass: A Phytochemical With Promising Antiviral Properties. *Front Nutr* **8**, 777576 (2022).

3. Mayo, B., Vázquez, L. & Flórez, A. B. Equol: A Bacterial Metabolite from The Daidzein Isoflavone and Its Presumed Beneficial Health Effects. *Nutrients* **11**, 2231 (2019).

4. Mackern-Oberti, J. P., Jara, E. L., Riedel, C. A. & Kalergis, A. M. Hormonal Modulation of Dendritic Cells Differentiation, Maturation and Function: Implications for the Initiation and Progress of Systemic Autoimmunity. *Arch Immunol Ther Exp (Warsz)* **65**, 123–136 (2016).

5. Zhen, Y., Krausz, K. W., Chen, C., Idle, J. R. & Gonzalez, F. J. Metabolomic and Genetic Analysis of Biomarkers for PPARα Expression and Activation. *Mol Endocrinol* **21**, 2136 (2007).

6. Zakaryan, H., Arabyan, E., Oo, A. & Zandi, K. Flavonoids: promising natural compounds against viral infections. *Arch Virol* **162**, 2539–2551 (2017).

7. Alexander, J. L. *et al.* The gut microbiota and metabolome are associated with diminished COVID-19 vaccine-induced antibody responses in immunosuppressed inflammatory bowel disease patients. *EBioMedicine* **88**, 104430 (2023).

8. Alvarez-Curto, E. & Milligan, G. Metabolism meets immunity: The role of free fatty acid receptors in the immune system. *Biochem Pharmacol* **114**, 3–13 (2016).

9. Lian, J. *et al.* The role of polyamine metabolism in remodeling immune responses and blocking therapy within the tumor immune microenvironment. *Front Immunol* **13**, 912279 (2022).

10. Cao, J. *et al.* Effects of altered glycolysis levels on CD8+ T cell activation and function. *Cell Death Dis* **14**, 1–13 (2023).

11. Patsoukis, N. *et al.* PD-1 alters T-cell metabolic reprogramming by inhibiting glycolysis and promoting lipolysis and fatty acid oxidation. *Nat Commun* **6**, 1–13 (2015).

12. Yoshida, S., Shime, H., Matsumoto, M., Kasahara, M. & Seya, T. Anti-oxidative amino acid L-ergothioneine modulates the tumor microenvironment to facilitate adjuvant vaccine immunotherapy. *Front Immunol* **10**, 436879 (2019).

13. Wu, H. liang *et al.* Targeting nucleotide metabolism: a promising approach to enhance cancer immunotherapy. *J Hematol Oncol* **15**, 45 (2022).

14. Tse, R. T. H., Wong, C. Y. P., Chiu, P. K. F. & Ng, C. F. The Potential Role of Spermine and Its Acetylated Derivative in Human Malignancies. *Int J Mol Sci* **23**, 1258 (2022).

15. Song, Z. *et al.* S-adenosylmethionine (SAMe) modulates interleukin-10 and interleukin-6, but not TNF, production via the adenosine (A2) receptor. *Biochimica et Biophysica Acta (BBA) - Molecular Cell Research* **1743**, 205–213 (2005).

**Table 8: Histology scoring**


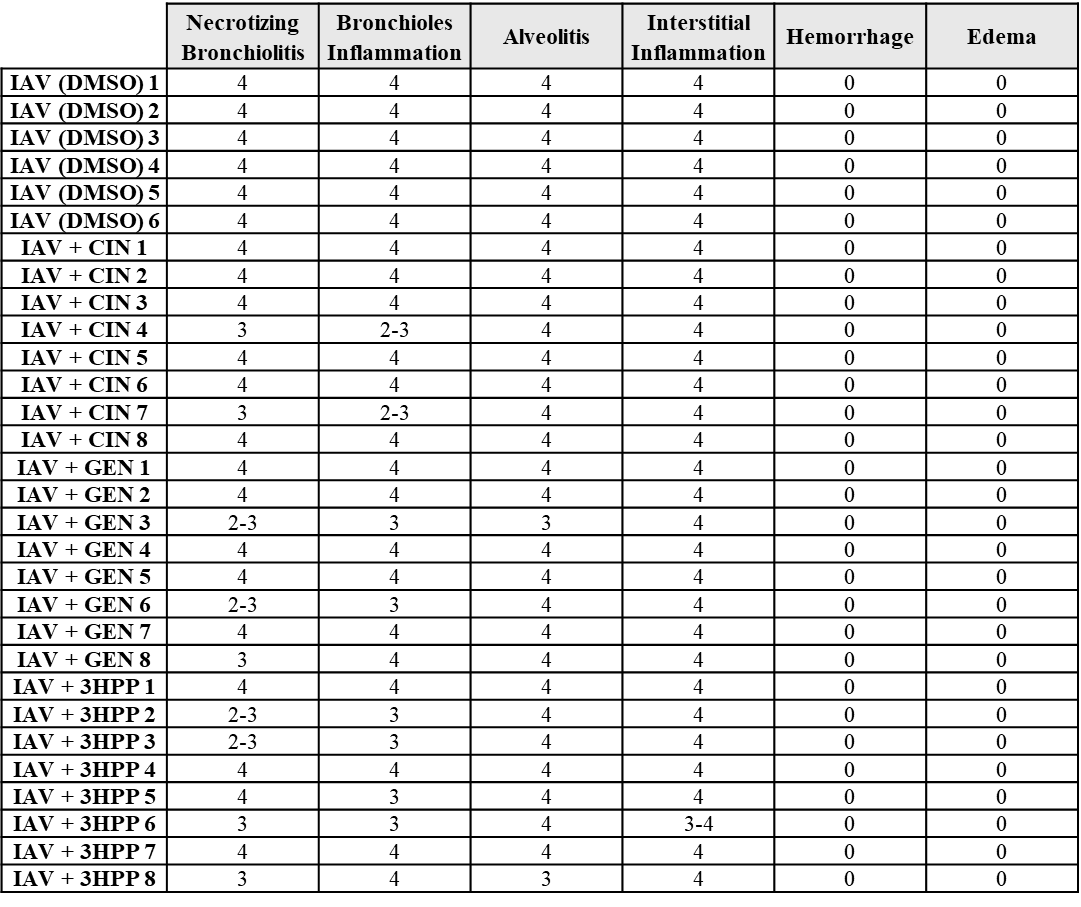


The severity of damage was scored on a scale ranging from **0** to **4**: **0** for none or very minor, **1** for mild, **2** for intermediate, **3** for moderately severe, and **4** for severe and widespread.
