# Supplementary figures and images for: Maternal probiotic exposure enhances CD8 T cell protective neonatal immunity and modulates offspring metabolome to control influenza virus infection
Source: Gut Microbes. 2024 Dec 22;17(1):2442526. doi: 10.1080/19490976.2024.2442526 (PMC12931718; doi:10.1080/19490976.2024.2442526)

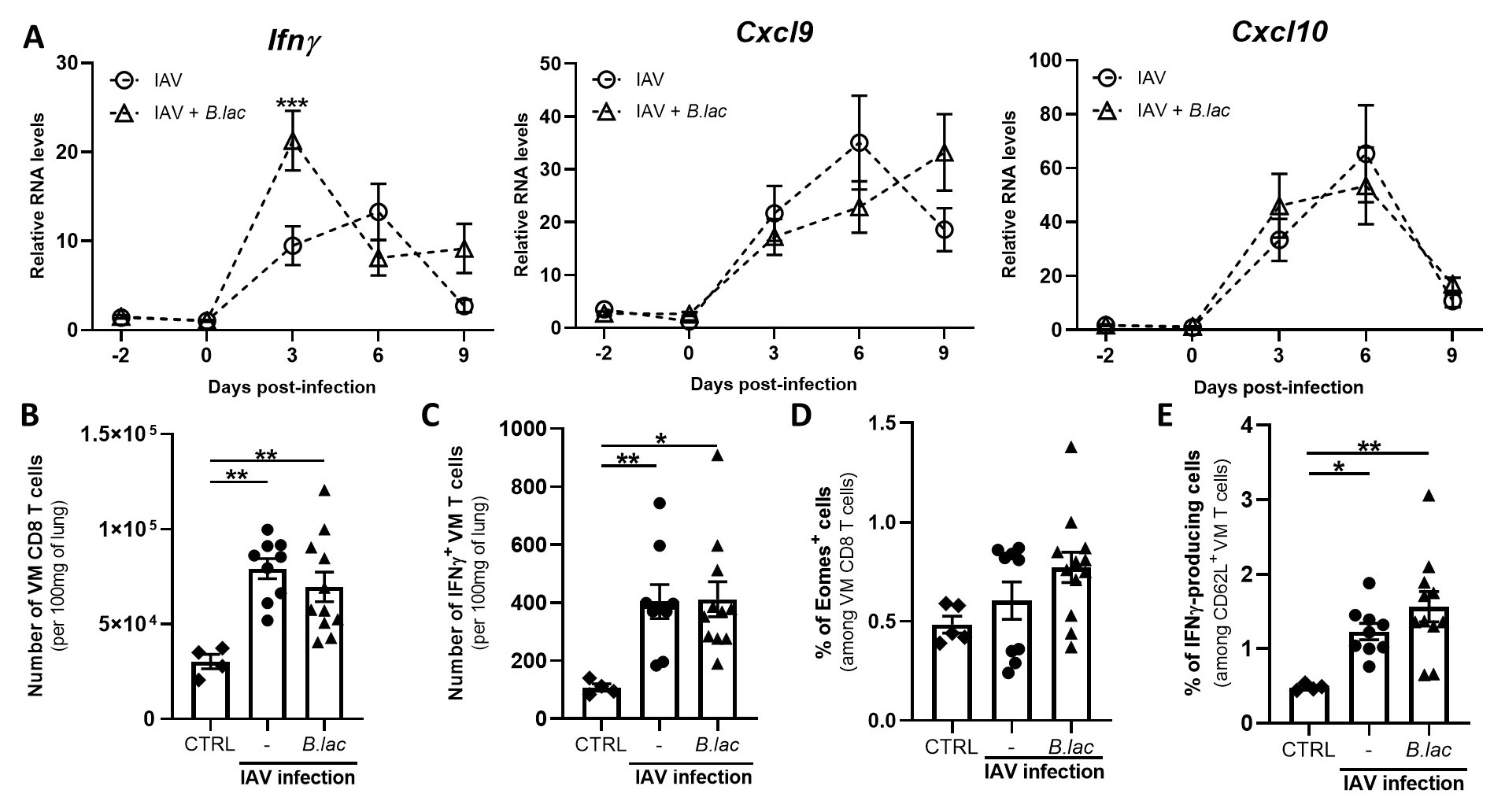

Supplement: Supplemental Material [file KGMI_A_2442526_SM7728.zip › kgmi-s-2024-1694-20241211215430/graphic/Figure S3.jpg]

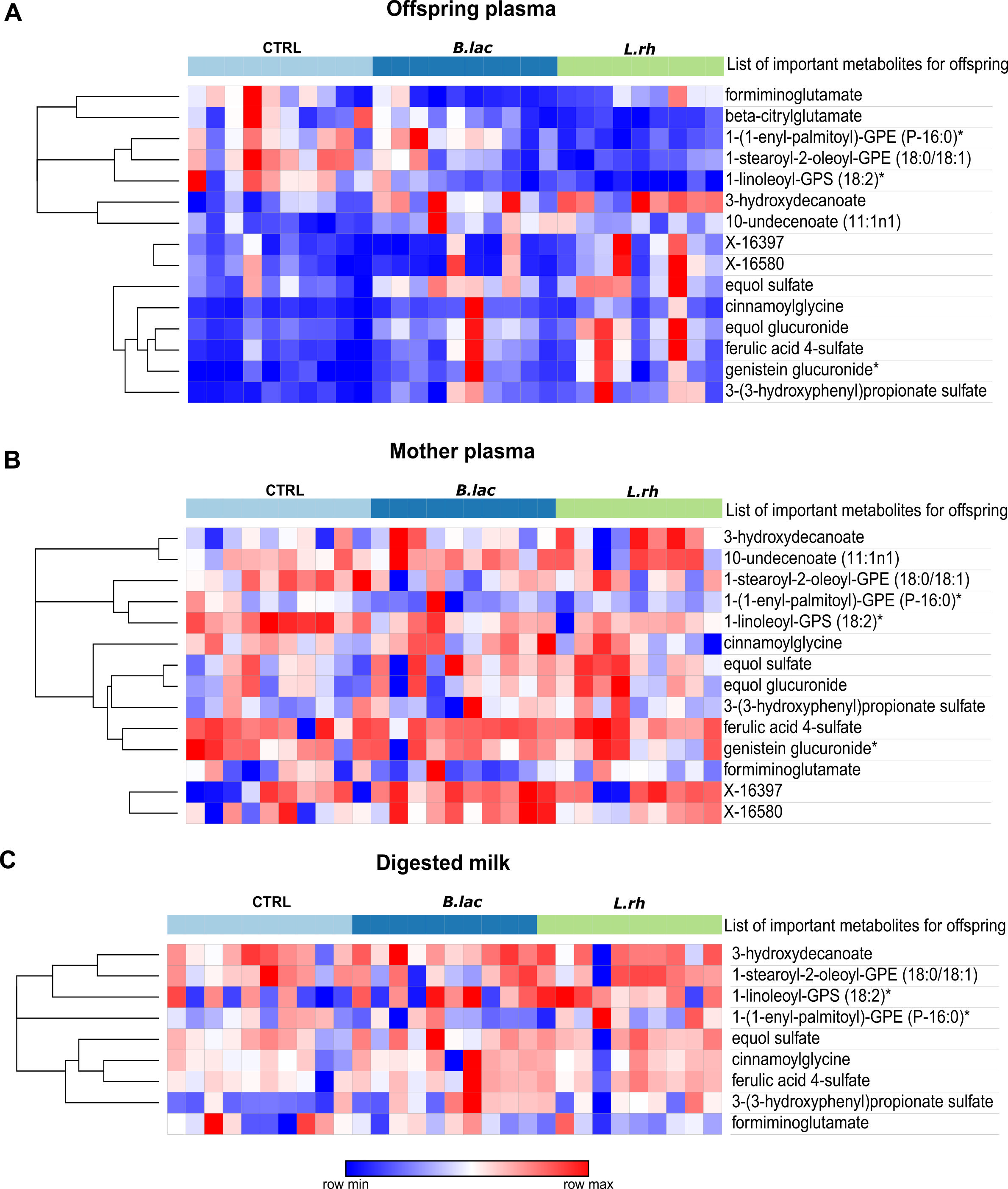

Supplement: Supplemental Material [file KGMI_A_2442526_SM7728.zip › kgmi-s-2024-1694-20241211215430/graphic/Figure S6.tiff]

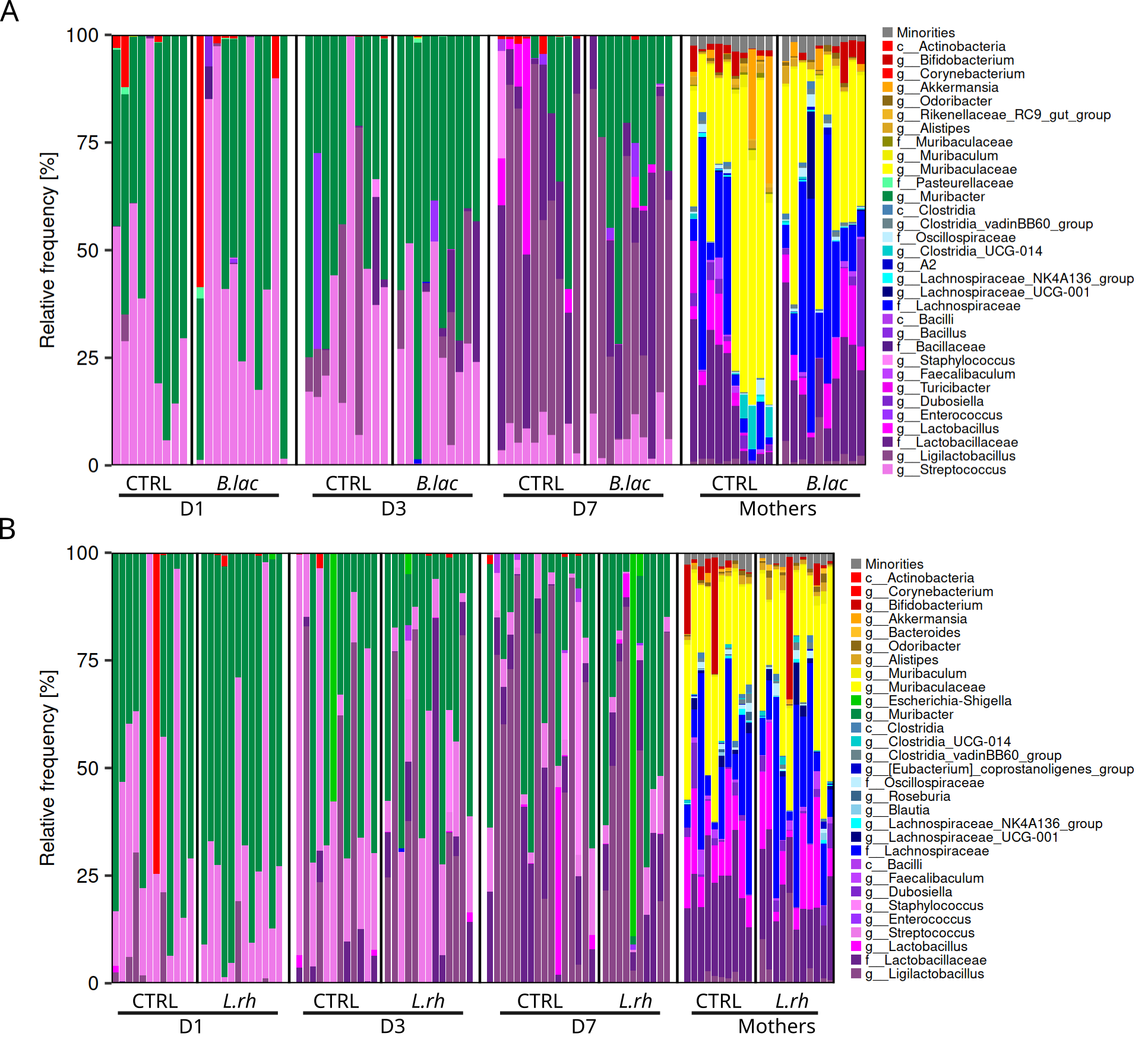

Supplement: Supplemental Material [file KGMI_A_2442526_SM7728.zip › kgmi-s-2024-1694-20241211215430/graphic/Figure S5.tiff]

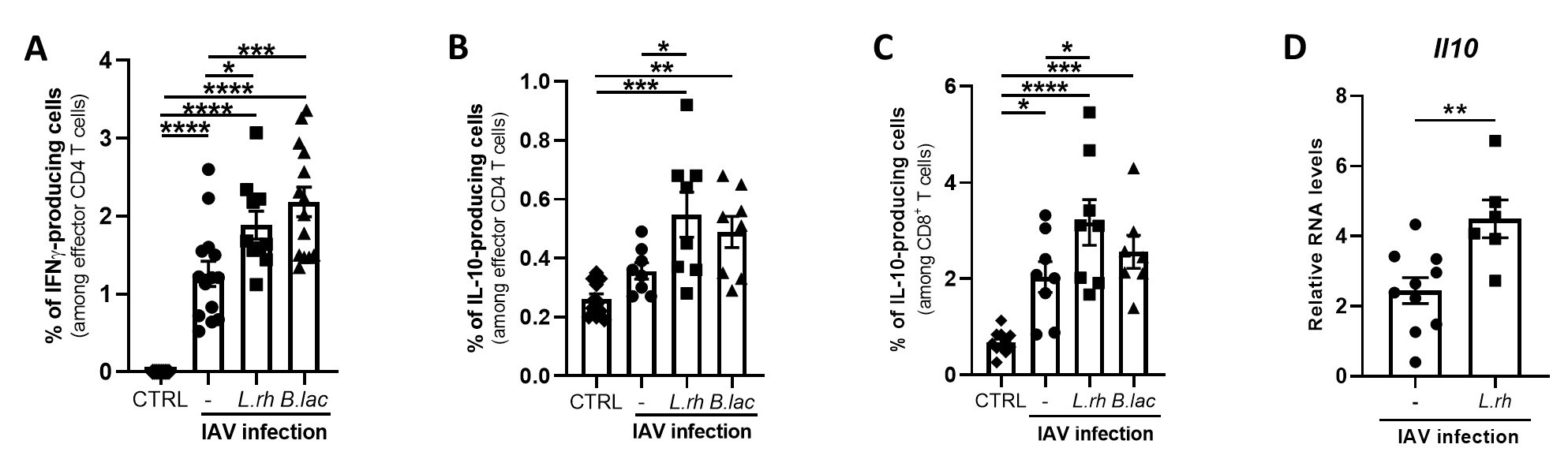

Supplement: Supplemental Material [file KGMI_A_2442526_SM7728.zip › kgmi-s-2024-1694-20241211215430/graphic/Figure S1.jpg]

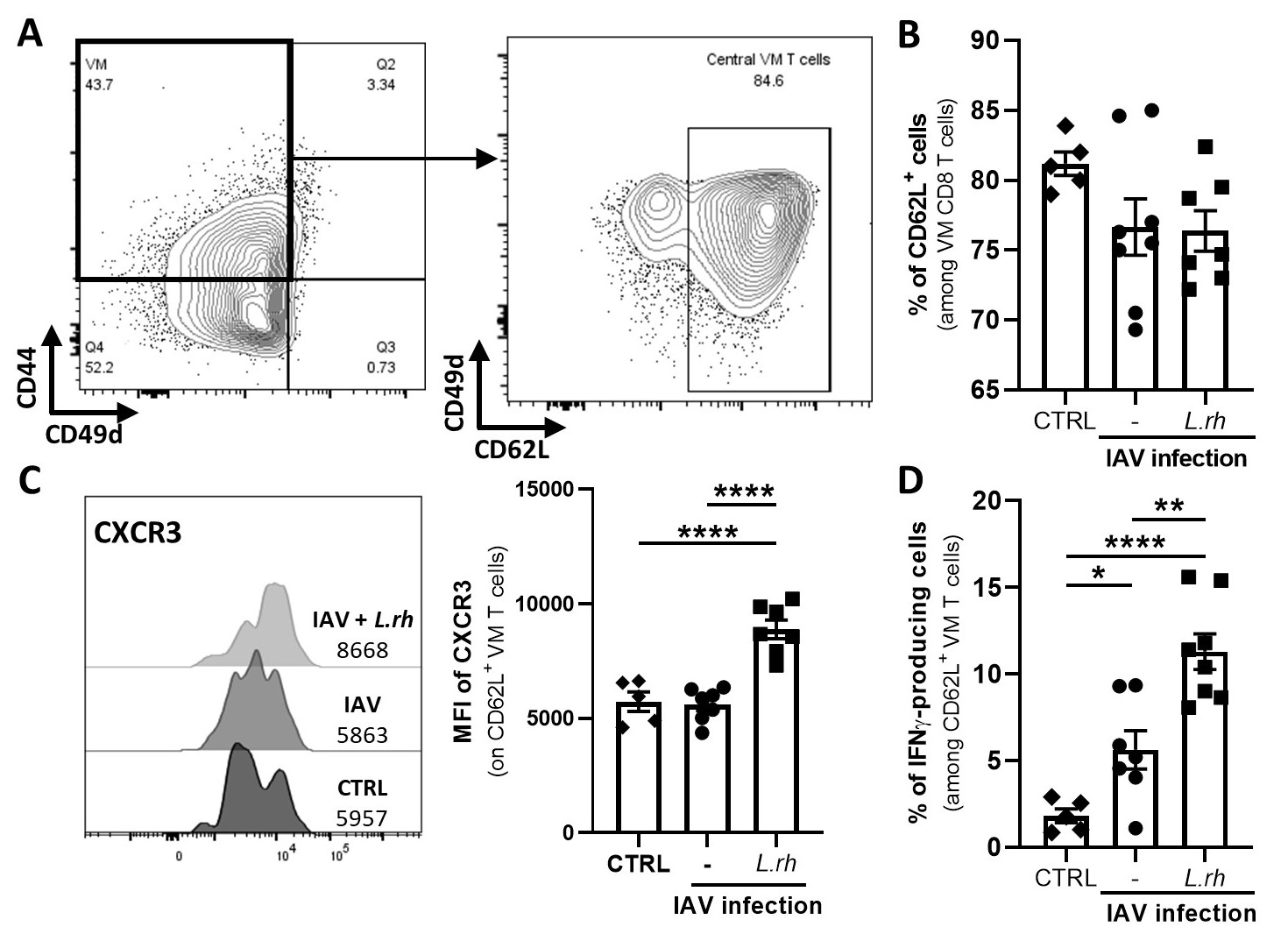

Supplement: Supplemental Material [file KGMI_A_2442526_SM7728.zip › kgmi-s-2024-1694-20241211215430/graphic/Figure S2.jpg]

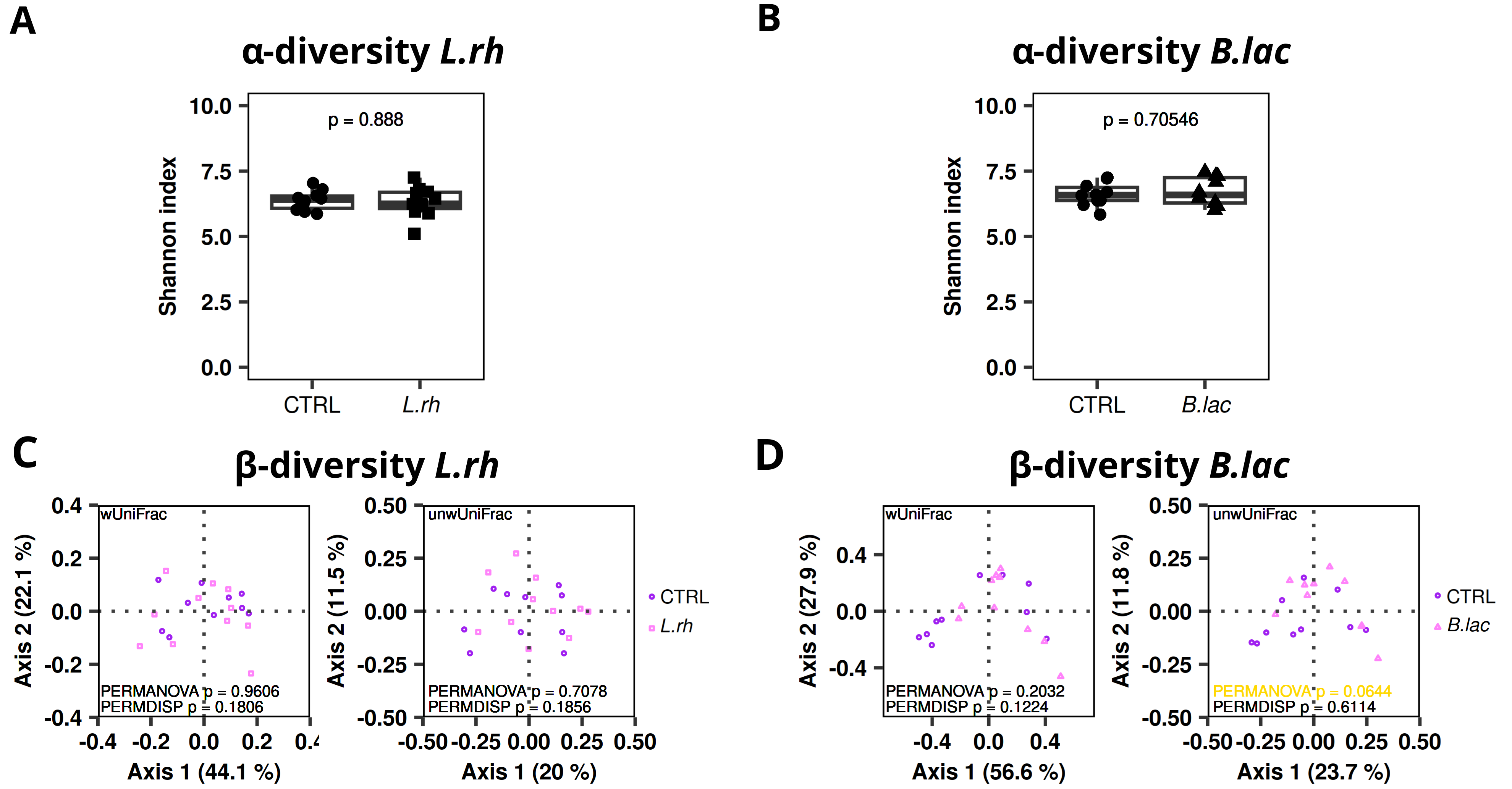

Supplement: Supplemental Material [file KGMI_A_2442526_SM7728.zip › kgmi-s-2024-1694-20241211215430/graphic/FigureS4.tiff]
